# Molecular Basis for poly(A) RNP Architecture and Recognition by the Pan2-Pan3 Deadenylase

**DOI:** 10.1016/j.cell.2019.04.013

**Published:** 2019-05-30

**Authors:** Ingmar B. Schäfer, Masami Yamashita, Jan Michael Schuller, Steffen Schüssler, Peter Reichelt, Mike Strauss, Elena Conti

**Affiliations:** 1 Department of Structural Cell Biology, MPI of Biochemistry, Munich, Germany; 2 cryoEM Facility, MPI of Biochemistry, Munich, Germany

**Keywords:** poly(A) tail, mRNA, RNP, PABP, Pab1, Pan2-Pan3, Ccr4-Not, deadenylase, RRM, cryoEM

## Abstract

The stability of eukaryotic mRNAs is dependent on a ribonucleoprotein (RNP) complex of poly(A)-binding proteins (PABPC1/Pab1) organized on the poly(A) tail. This poly(A) RNP not only protects mRNAs from premature degradation but also stimulates the Pan2-Pan3 deadenylase complex to catalyze the first step of poly(A) tail shortening. We reconstituted this process *in vitro* using recombinant proteins and show that Pan2-Pan3 associates with and degrades poly(A) RNPs containing two or more Pab1 molecules. The cryo-EM structure of Pan2-Pan3 in complex with a poly(A) RNP composed of 90 adenosines and three Pab1 protomers shows how the oligomerization interfaces of Pab1 are recognized by conserved features of the deadenylase and thread the poly(A) RNA substrate into the nuclease active site. The structure reveals the basis for the periodic repeating architecture at the 3′ end of cytoplasmic mRNAs. This illustrates mechanistically how RNA-bound Pab1 oligomers act as rulers for poly(A) tail length over the mRNAs’ lifetime.

## Highlights

• Oligomerization of PABP on the poly(A) tail creates a series of consecutive arches
• Pan2-Pan3 deadenylase recognizes the oligomerized state of poly(A)-bound PABP
• The dimerization interface of juxtaposed PABPs creates the Pan2-Pan3 docking site
• The poly(A) RNP arches are flexible and moldable by the interacting proteins

## Graphical Abstract

**Figure:**
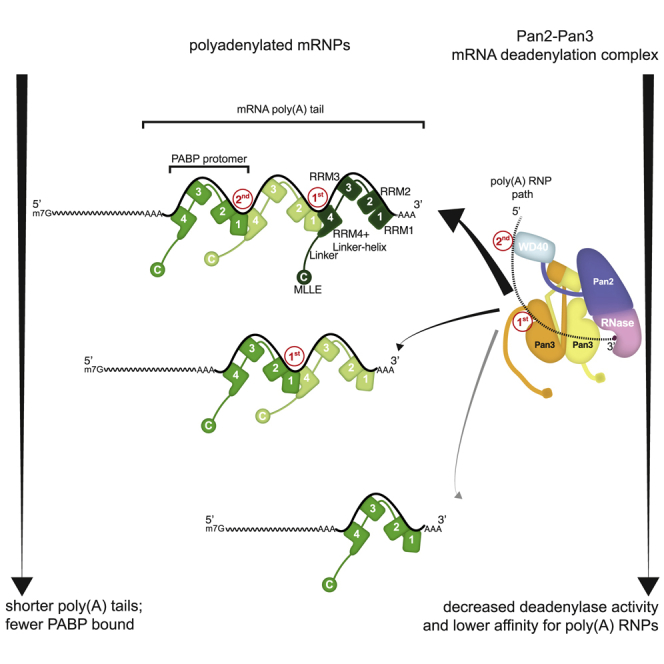

## Teaser

A structural view of the interplay between a deadenylase and its substrate reveals how oligomers of poly(A) binding protein are recognized by the Pan2-Pan3 deadenylase and how these RNA-bound oligomers act as rulers for poly(A) tail length

## Introduction

The discovery of the poly(A) tail as the specialized sequence feature at the 3′ end of eukaryotic mRNAs dates back nearly fifty years (Darnell et al., 1971, Edmonds et al., 1971, Lee et al., 1971). Virtually all eukaryotic mRNAs (with the notable exception of mammalian replication-dependent histone mRNAs) feature a string of adenosines that are added to the 3′ end of the nascent transcript by the nuclear poly(A) polymerase (Edmonds, 2002). The length of the poly(A) tail synthesized upon transcription termination varies in different species, from about 90 nucleotides in yeast to 200 to 250 in mammals (reviewed in Eckmann et al., 2011). The crucial roles of the poly(A) tail in mRNA export, translation, and decay, however, are universally conserved and largely mediated by its specific binding partners, the poly(A)-binding proteins (PABPs) (reviewed in Eliseeva et al., 2013, Kühn and Wahle, 2004).

Cytoplasmic PABPs are predominant components of messenger ribonucleoprotein particles (mRNPs) (Singh et al., 2015). Evidence over the years has resulted in the notion that yeast Pab1 and its mammalian orthologue poly(A) binding protein cytoplasmic 1 (PABPC1) coat the 3′ end of cytoplasmic mRNAs, packaging them into poly(A) RNPs (reviewed in Eliseeva et al., 2013, Kühn and Wahle, 2004). Cytoplasmic PABPs are very abundant proteins (μM concentration), bind poly-adenylate RNAs with very high affinity (nM Kd), and organize the poly(A) tail into a repeating pattern with a periodicity of about 27 nucleotides, the footprint of a single PABP (Baer and Kornberg, 1980, Görlach et al., 1994, Kühn and Pieler, 1996, Sachs et al., 1987). The RNA-binding properties of Pab1 and PABPC1 lie in their N-terminal region, which consists of four consecutive RNA-recognition motif (RRM) domains (reviewed in Eliseeva et al., 2013, Kühn and Wahle, 2004). The two N-terminal RRM1 and RRM2 domains function as a single module that is essentially responsible for the adenosine-binding specificity and affinity characteristic of the full-length protein (Kühn and Pieler, 1996, Sachs et al., 1987). Structural studies have shown that this module also provides directionality to poly(A) binding, with the RNA being recognized in a 3′-to-5′ polarity from RRM1 to RRM2 (Deo et al., 1999). Although it has been generally assumed that RRM3 and RRM4 may form a similar structural module, it is nevertheless clear that they are functionally different, at least in terms of RNA-binding properties (Kühn and Pieler, 1996, Deardorff and Sachs, 1997, Yao et al., 2007). The RRM domains also contribute to protein-protein interactions. RRM2, for example, binds a component of the translation initiation machinery (eIF4G) (Safaee et al., 2012). The main protein-binding platform of PABP, however, resides in the C-terminal region of the molecule (reviewed in Xie et al., 2014). At the very C terminus, the Mademoiselle (MLLE) domain recognizes short sequence motifs (known as PAM2) that are present, for example, in factors involved in translation termination (eRF3) (Hoshino et al., 1999), mRNA deadenylation (Pan3) (Siddiqui et al., 2007), and, in the case of the metazoan proteins, micro-RNA-mediated gene silencing (TNRC6/GW182) (Fabian et al., 2009). Finally, the linker between the last RRM and the MLLE domains plays an important role in mediating the cooperative binding of multiple PABP molecules to the poly(A) tail (Simón and Séraphin, 2007). The poly(A) RNA-dependent oligomerization of Pab1 and PABPC1 is a hallmark of the repetitive organization of the poly(A) RNP.

The poly(A) RNP is one of the most dynamic features of an mRNP. During the lifetime of the transcript, the poly(A) tail is gradually shortened, eventually leading to mRNA degradation (reviewed in Eckmann et al., 2011, Jalkanen et al., 2014). PABP plays an interesting dichotomous role in this context. On the one hand, it physically protects the 3′ end of the transcript from unspecific degradation (Ford et al., 1997). On the other hand, it is required for poly(A) tail shortening (Caponigro and Parker, 1995, Sachs and Davis, 1989). Indeed, the cytoplasmic PABP interacts with and stimulates Pan2-Pan3, the deadenylase complex that trims long poly(A) tails (Uchida et al., 2004). In addition, PABP interacts with Ccr4-Not, the deadenylase complex that triggers mRNA decay (Daugeron et al., 2001, Tucker et al., 2001, Webster et al., 2018).

Genetic and biochemical studies in yeast and human cells have shown that deadenylation proceeds in distinct phases (Decker and Parker, 1993, Yamashita et al., 2005). *In vivo*, S. cerevisiae Pan2-Pan3 performs the initial trimming of the 3′ end to about 50 to 70 adenosines and can continue shortening until the poly(A) tract reaches a critical length of 25 to 40 nucleotides (Beilharz and Preiss, 2007, Brown and Sachs, 1998, Mangus et al., 2004). Deadenylation then transitions from this initial slow phase to a second phase that commits the mRNA to turnover (Decker and Parker, 1993). In the turnover phase, Ccr4-Not erodes the poly(A) tail even further to a short poly(A), rendering the transcript susceptible to decapping followed by 5′-3′ degradation via Xrn1 and/or to 3′-5′ degradation by the exosome-Ski complex (Daugeron et al., 2001, Decker and Parker, 1993, Tucker et al., 2001). mRNA transcripts also exhibit bimodal deadenylation kinetics in human cells, although the polyadenylated intermediates are generally longer and more heterogeneous (Yamashita et al., 2005). The molecular mechanisms underpinning the transition between the Pan2-Pan3 and Ccr4-Not complexes and the basis for their different substrate preferences are unknown. How Pan2-Pan3 specifically recognizes early poly(A) RNPs and why it stops after shortening them to a discrete size is currently not understood. Furthermore, insights into the structure of the poly(A) RNP have remained largely elusive, despite its central role in the life of a mature mRNA. Here, we address these questions by employing biochemical and structural approaches.

## Results

### Reconstitution of Poly(A) RNP Deadenylation Recapitulates Yeast Poly(A) Tail-Length Distribution

**Figure 1:**
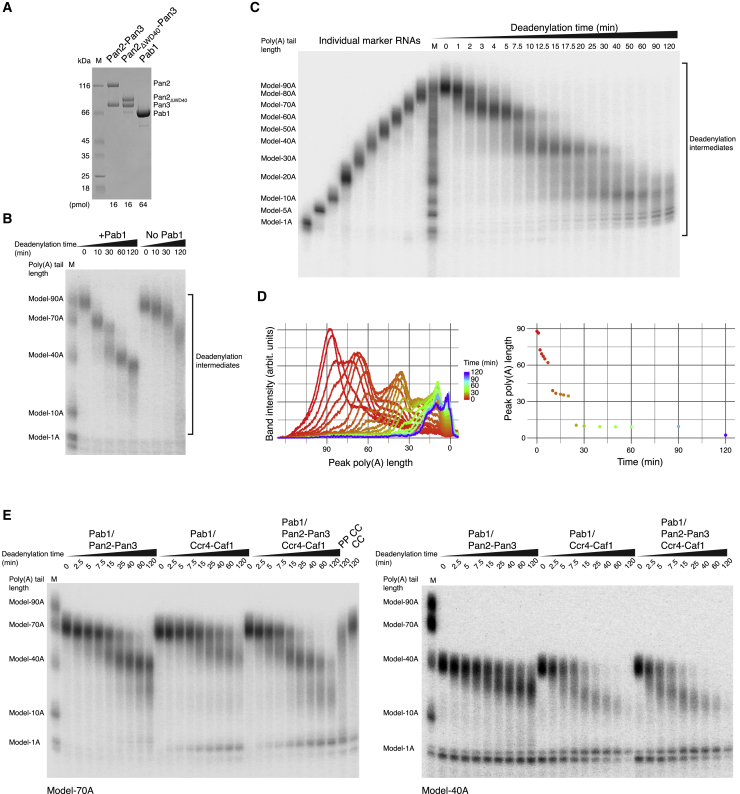
Poly(A) RNP Features Underpinning Pan2-Pan3 Deadenylation Activity (A) Recombinant proteins used in deadenylation reactions. 12.5% SDS-PAGE gel visualized via Coomassie staining showing the purified recombinant proteins used in the assays: wild-type S. cerevisiae Pan2-Pan3 (Schäfer et al., 2014), Pan2ΔWD40-Pan3 (Figures 5, 6, and S6B), and Pab1 (Schäfer et al., 2014). M indicates the lane with size markers (in all gels of the manuscript). (B) *In vitro* deadenylation activity of Pan2-Pan3 is stimulated by Pab1. A model-90A RNA was radioactively labeled at the 5′ end and incubated with wild-type Pan2-Pan3 in the absence or presence of Pab1 (in a 1:3 RNA:protein ratio, indicated by “no Pab1” and “+Pab1,” respectively; see also Figure S6B). Samples were withdrawn at indicated time points and the reactions were stopped. The samples were analyzed on a 6% denaturing Urea-PAGE gel followed by phosphorimaging. (C) *In vitro* deadenylation of a yeast 90A RNP substrate by Pan2-Pan3 results in a phased poly(A) tail distribution. The same 5′-labeled model-90A RNA described in (B) was mixed with Pab1 (in a 1:3 RNA:protein ratio) and incubated with wild-type Pan2-Pan3 for 2 h. At indicated time points, samples were withdrawn and the reaction was stopped (see also Figure 5B). The samples of the deadenylation time course (right lanes) and the molecular weight markers (left lanes) were analyzed on a 6% denaturing Urea-PAGE gel followed by phosphorimaging. The recombinant proteins used in the assays are shown in (A). (D) Quantitation of the *in vitro* deadenylation experiment reveals increased activity of Pan2-Pan3 on longer poly(A) RNPs. The raw data from (C) were quantified by densitometric analysis of each gel lane (left) and summarized by plotting the poly(A) length at peak intensity for each time point (right). See also Figure S1A and Table S2. (E) Deadenylation time course of a model-70A RNP (1:2 RNA:Pab1 ratio, left panel) and a model-40A RNP (1:1 RNA:Pab1 ratio, right panel) upon addition of either Pan2-Pan3 or a Caf1-Ccr4 complex (Basquin et al., 2012) or both. The reactions were stopped at the indicated time points and analyzed on a 6% Urea-PAGE followed by phosphorimaging. On the left panel, the last two lanes mark 120-min-long control reactions in the absence of Pab1 with both Pan2-Pan3 and Caf1-Ccr4 (PP CC) or only with Caf1-Ccr4 (CC). See also Figure S1E.

We first established a biochemical system to reproduce the *in vivo* properties of yeast poly(A) RNP deadenylation in an *in vitro* setting with purified components (Figures 1A, 1B, and 1C). To mimic the poly(A) tail of a newly synthesized yeast mRNP entering the cytoplasm, we produced a model RNA substrate with a stretch of 90 adenosines (A) at the 3′ end by *in vitro* transcription (model-90A RNA). Given the Pab1 RNA-binding footprint of 27 nt (Baer and Kornberg, 1983, Sachs et al., 1986), three protomers are expected to cover a 90A RNA. We thus reconstituted a model-90A RNP using a three-fold molar excess of Pab1 and added recombinant Pan2-Pan3 to initiate deadenylation. The experiment resulted in the rapid and prominent accumulation of ∼70A intermediate fragments that were then converted into ∼40A fragments, eventually leading to a rather slow and weak accumulation of 10A RNA products (Figure 1C). The pattern of RNA intermediates in this reconstituted system was dependent on and stimulated by the presence of Pab1 (Figure 1B) and suggested that Pan2-Pan3 degrades the poly(A) tail in bursts that are connected to the stepwise removal of Pab1 molecules.

**Figure S1:**
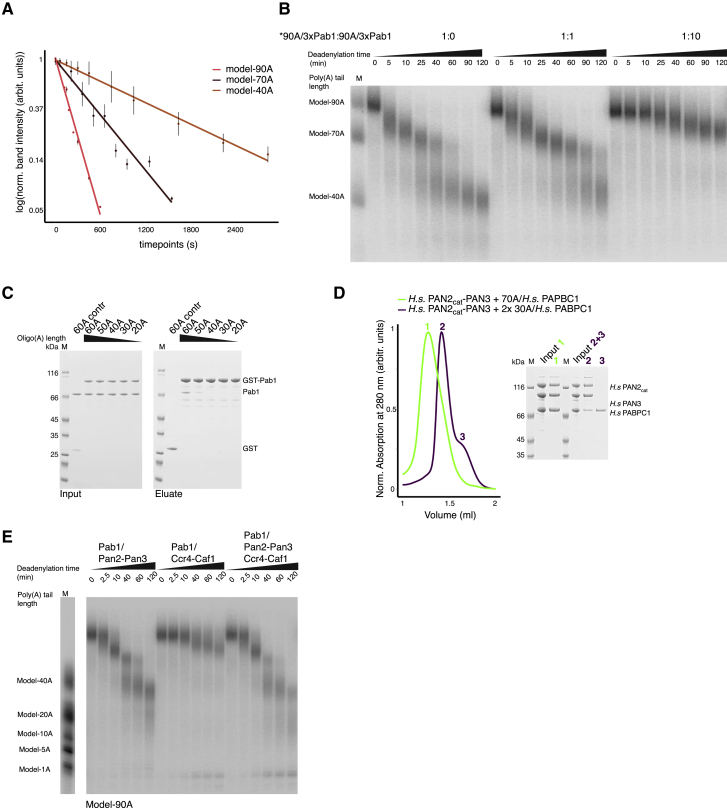
Yeast and Human Pan2-Pan3 Complexes Preferentially Bind and Deadenylate poly(A) RNPs, Related to Figure 2 (A) Pan2-Pan3 is more active on longer poly(A)/Pab1 RNPs in *in vitro* deadenylation experiments. Three phosphorimages of UREA-PAGE gels of Pan2-Pan3 mediated deadenylation assays with a model-90A RNP as educt (similar and including Figure 1C) were quantified by densitometry. After normalization and natural log-transformation, median band intensities per time point for the model-90A RNP as well as the two intermediates, the model-70A RNP and the model-40A RNP are plotted. In all three cases the respective timepoint 0 corresponds to the timepoint in the assay with the most intense densitometric reading for the respective model substrate. Vertical lines represent the standard deviation interval at each particular time-point. The trend lines are the curves fitted to determine the half-lives of the respective model poly(A) RNPs (see Table S2). (B) Poly(A) tail length preference of Pan2-Pan3. The 5′-labeled 90A RNP was subjected to deadenylation in the presence of increasing amounts of unlabeled 90A RNP. The ratios between radioactive (hot) 90A and not radioactive (cold) 90A used in the competition assays are specified above each time course (each RNA substrate was incubated independently with Pab1 prior to the reaction). At indicated time points samples were taken and analyzed on a 6% Urea-PAGE followed by phosphorimaging. (C) Interaction of multiple Pab1 protomer on longer oligo(A) RNAs. We incubated GST-tagged and untagged Pab1 in the presence of oligo(A) RNAs of different length, ranging between 20A and 60A, and tested the interaction in pull-down assays with glutathione-agarose beads. In this experiment, an increase from 40A to 60A was required to observe significant Pab1-Pab1 co-precipitation. The Coomassie-stained 4%–12% SDS-PAGE gels show the input (left) and the pulled-down protein precipitates (right). (D) The interaction of human PAN2-PAN3 and PABPC1 depends on poly(A) tail length. Size-exclusion chromatography (SEC) assays carried out with a PAN2cat-PAN3 catalytically inactive mutant (D1087A, orthologues to D1020A in S. cerevisiae). In case of the comigration experiments of PAN2cat-PAN3 and 30A/PABPC1 we used 2x molar excess of the RNP to ensure a comparable concentration of binding sites and bases present as in the longer poly(A) RNPs. Left panel: overlays of the chromatograms. Right panel: Coomassie-stained 4%–12% SDS–PAGE gels with samples from the input and peak fractions. (E) Deadenylation time course of a model-90A RNP (1:3 RNA:Pab1 ratio) upon addition of either Pan2-Pan3 or a Caf1-Ccr4 complex (Basquin et al., 2012), or both. The reactions were stopped at the indicated time points and analyzed on a 6% Urea-PAGE followed by phosphorimaging.

**Figure 2:**
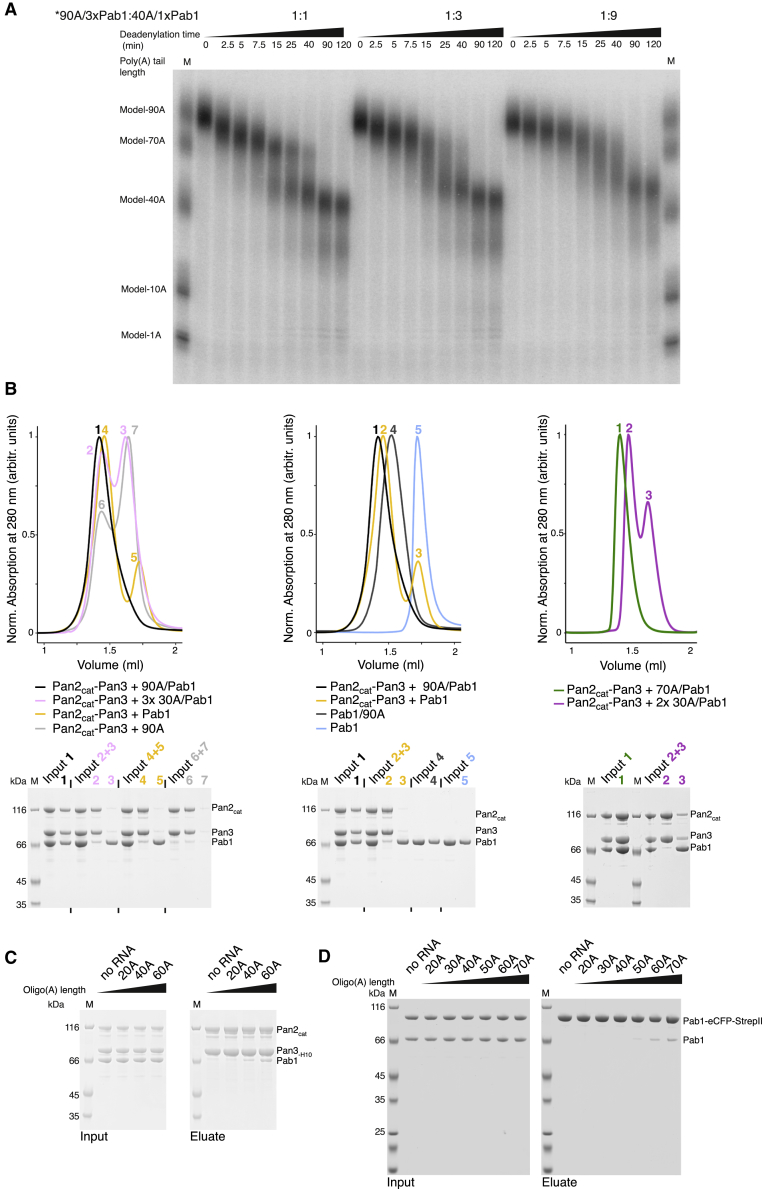
Impact of poly(A) Tail Length on the Interaction with Pan2-Pan3 (A) Poly(A) tail length preference of Pan2-Pan3. The 5′-labeled 90A RNP was subjected to deadenylation in the presence of increasing amounts of unlabeled 40A RNP (for 90A RNP versus 90A RNP competition experiment, see Figure S1B). The ratios between radioactive (hot, indicated by an asterisk) 90A and not radioactive (cold) 40A used in the competition assays are indicated above each time course (each RNA substrate was incubated independently with Pab1 prior to the reaction). At indicated time points, samples of the reactions were taken and analyzed on a 6% Urea-PAGE followed by phosphorimaging. (B) The interaction of Pan2-Pan3 and Pab1 depends on poly(A) tail length. SEC assays carried out with a Pan2cat-Pan3 catalytically inactive mutant (D1020A). In case of the comigration experiments of Pan2cat-Pan3 and 30A/Pab1, we used 3× or 2× molar excess of the RNP to ensure a comparable concentration of binding sites and bases present as in the longer poly(A) RNPs. Upper panel: overlays of the chromatograms. Lower panel: Coomassie-stained 4%–12% SDS–PAGE gels with samples from the input and peak fractions. (C) RNA-dependent co-precipitation of Pab1 and Pan2cat-Pan3 in Ni-NTA pull-down assays. Oligo(A) RNAs were mixed with Pab1 (2× molar excess) and incubated with His-tagged Pan2cat-Pan3. The Coomassie-stained 4%–12% SDS-PAGE gels show the input (left) and the pulled-down protein precipitates (right). (D) Interaction of multiple Pab1 protomers on longer oligo(A) RNAs. We incubated C-terminal eCFP-StrepII-tagged and untagged Pab1 in the presence of oligo(A) RNAs of different length, ranging between 20A and 70A, and tested the interaction in pull-down assays with Strep-Tactin beads. In this experiment, an increase from 40A to 60A was required to observe significant Pab1-Pab1 co-precipitation. The Coomassie-stained 4%–12% SDS-PAGE gels show the input (left) and the pulled-down protein precipitates (right).

In the deadenylation assay, the degradation rates decreased gradually as the substrate was shortened (Figure 1C). The half-life of the model-90A RNA was three-fold shorter than that of the 70A intermediate fragment and roughly nine-fold shorter than that of the ∼40A fragments, indicating that longer RNPs were degraded faster (Figure S1A and Table S2). To analyze the apparent preference of Pan2-Pan3 for longer poly(A) RNPs, we employed a substrate competition assay. We incubated Pan2-Pan3 with a labeled 90A RNP substrate and challenged it with increasing amounts of either unlabeled 40A RNP (Figure 2A) or unlabeled 90A RNP (Figure S1B). In this assay, the more unlabeled 90A poly(A) RNP was present in the reaction, the less degradation of the radioactively labeled 90A RNP was observed. When challenging the reaction with unlabeled 40A RNP, however, there was essentially no inhibitory effect on deadenylation of the labeled 90A RNP (Figure 2A), demonstrating that Pan2-Pan3 has a clear preference for longer poly(A) RNPs.

The sequential action of Pan2-Pan3 and Ccr4-Not deduced from *in vivo* experiments has suggested that the two deadenylase complexes have different substrate preferences (Decker and Parker, 1993, Tucker et al., 2001). To recapitulate their interplay in an *in vitro* setting, we challenged Pan2-Pan3 by adding recombinant catalytic subunits of the Ccr4-Not complex, Caf1-Ccr4 (which have been shown to act on poly(A) RNAs and poly(A) RNPs, respectively; Webster et al., 2018, Yi et al., 2018). Nuclease assays with either individual or both deadenylases showed that Pan2-Pan3 activity indeed dominated in the case of a 90A and a 70A RNP substrate, whereas Caf1-Ccr4 activity dominated with a 40A RNP substrate (Figure 1E and Figure S1E). Interestingly, the combination of Pan2-Pan3 and Caf1-Ccr4 resulted in an overall more productive deadenylation of the full substrate than either enzyme alone (Figure 1E and Figure S1E). Both Pan2-Pan3 and the combination of Pan2-Pan3 plus Ccr4-Not deadenylate long poly(A) RNPs with comparable rates. This indicates that the observed increase in overall deadenylation is a product of a synergistic effect between both deadenylases rather than a result of a mere increase in total deadenylase concentration (Figure S1E). Such cumulative effect is in line with the complementary specificities of each enzyme in acting preferentially at different deadenylation stages and, in turn, suggests how the sequential series of these enzymatic activities may be more efficient than either one alone. Finally, the activities of both S. cerevisiae deadenylases, Pan2-Pan3 (Figure 1B), but also Caf1-Ccr4 (Figure 1E, lane CC) are stimulated by Pab1. This aligns well with similar observations recently reported for the human and S. pombe proteins (Webster et al., 2018, Yi et al., 2018).

We conclude that Pan2-Pan3 can effectively degrade a poly(A) tail long enough to span three Pab1 protomers to a shorter tail spanning two Pab1 protomers but is ineffective when the poly(A) tail has been shortened to span a single Pab1 (i.e., in the 30A to 40A range). The *in vitro* system reconstituted with purified components can thus reproduce the phased length distribution of poly(A) tail lengths in yeast, and it also recapitulates the *in vivo* substrate specificity of the two deadenylase complexes.

### The Pan2-Pan3 Deadenylase Binds Poly(A) RNPs with Two or More Pab1s

The observation that Pan2-Pan3 is more active on long poly(A) tails raises the question of whether the deadenylation properties of this complex are connected to its substrate-binding properties. The current view is that poly(A) RNP substrates are recognized by the N-terminal domain of Pan3: the Zinc-finger domain has been shown to interact with a poly(A) RNA (Wolf et al., 2014) and the PAM2 motif binds to the C-terminal domain of the poly(A) binding proteins (Siddiqui et al., 2007). The caveat, however, is that these interactions are weak (Kd in the μM range; Siddiqui et al., 2007, Wolf et al., 2014). We assessed the binding of Pan2-Pan3 by size exclusion chromatography (SEC) using a catalytically inactive version of the deadenylase complex that we had previously characterized (Pan2 Asp1020Ala mutant, Pan2cat; Schäfer et al., 2014). In SEC co-migration experiments, we could not detect a significant association between Pan2cat-Pan3 and Pab1 (Figure 2B, left panel, peaks 4 and 5 in yellow) or between Pan2cat-Pan3 and a 90A RNA (left panel, peaks 6 and 7 in light gray). In contrast, Pan2cat-Pan3 and a Pab1-90A RNP co-eluted as a single peak (left panel, peak 1 in black) with a smaller retention volume than either Pan2cat-Pan3 alone, Pab1 alone, or Pab1/90 RNP alone (Figure 2B, central panel, compare peak 1 in black with peaks 2 in yellow, 3 in yellow, and 4 in dark gray). These results indicate the formation of a stable Pan2-Pan3-Pab1/90A RNP assembly.

Next, we used the SEC assays to assess whether the length of the poly(A) RNA has an effect on complex formation. Strikingly, a 30A RNP (purified after mixing a 30A RNA and Pab1 in a 1:1 molar ratio) did not associate with Pan2cat-Pan3 (Figure 2B, left panel, peaks 2 and 3 in pink), whereas a 70A RNP (purified after mixing a 70A RNA and Pab1 in a 1:2 molar ratio) co-eluted with Pan2cat-Pan3 as a stable assembly (Figure 2B, right panel, peak 1 in green, compare with the individual smaller subcomplexes detected with a 30A RNP sample, peaks 2 and 3 in violet). We confirmed the RNA-length dependency of the interaction between Pan2cat-Pan3 and poly(A) RNPs by carrying out pull-down assays with oligo(A) RNAs of different lengths (Figure 2C). Using nickel-chelating beads, a His-tagged version of Pan2cat-Pan3 significantly co-precipitated with untagged Pab1 when the length of the RNA increased from 40A to 60A (Figure 2C). Given the Pab1 footprint (Baer and Kornberg, 1983, Sachs et al., 1986), an increase from 40A to 60A is also expected to allow binding of two Pab1 protomers per RNA molecule. To validate this prediction, we tested the oligomerization properties of Pab1 in pull-down assays. Using Strep-Tactin Sepharose beads, we found that an increase from 40A to 60A RNA was required to observe significant co-precipitation of an eCFP-StrepII-tagged version of Pab1 with untagged Pab1 (Figures 2D and S1C).

We concluded that Pan2-Pan3 does not stably interact with short poly(A) RNPs containing a single Pab1 but requires longer poly(A) RNPs containing two or more Pab1 protomers. At the biochemical level, these properties appear to be evolutionarily conserved from yeast to human, as we observed a similar pattern using recombinant human PAN2cat-PAN3 and PABPC. In SEC assays, human PAN2cat-PAN3 co-eluted in a single peak with a 70A RNP but not with a 30A RNP (Figure S1D). To understand the recognition mechanisms at the molecular level, we set out to determine the cryo-EM structure of Pan2cat-Pan3 complex with a long poly(A) RNP.

### The Pan2-Pan3 Complex Adopts Similar Conformations in the 90A/Pab1 Bound and Unbound State

**Figure S2:**
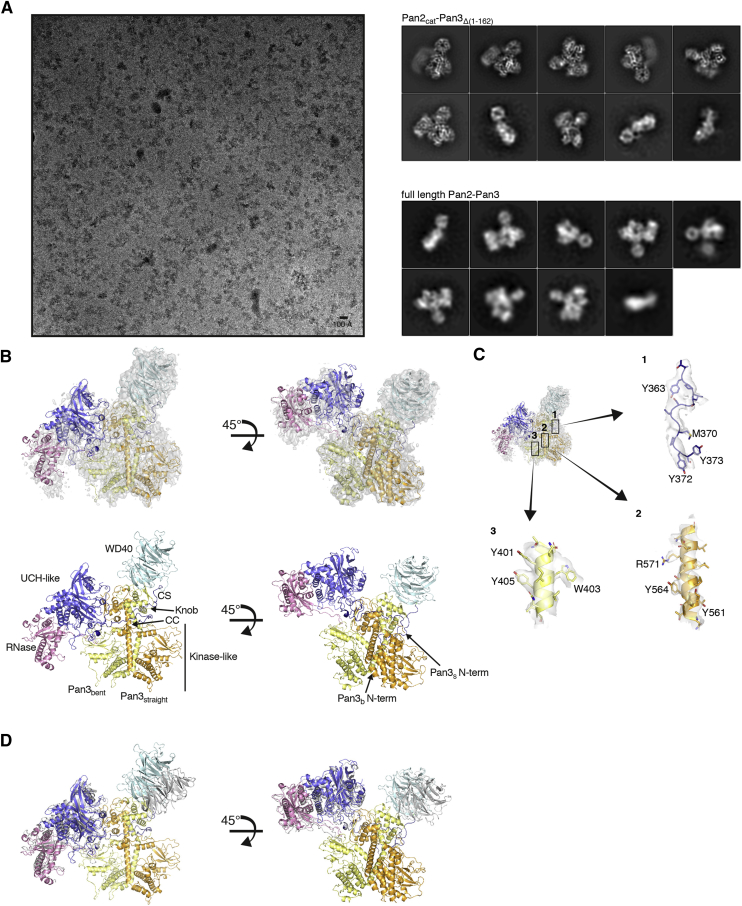
Structural Characterization of the Apo Pan2-Pan3 Complex by cryoEM, Related to Figure 3 (A) Representative micrograph and 2D class averages of the Pan2cat-Pan3Δ(1-162) dataset left and top right panels, respectively) and 2D class averages of a full-length Pan2-Pan3 dataset (bottom right panel). A circular mask of 210 Å diameter was used in the 2D classification. No additional density for the Pan3 N termini can be observed in the reference free 2D classes of the full-length Pan2-Pan3 data. (B) 3D reconstruction of the Pan2cat-Pan3Δ(1-162) data with rigid body fitted models of Pan2-Pan3. The coloring scheme resembles that outlined in Figure 3A for Pan2-Pan3. Briefly the Pan2 WD40 domain is colored in cyan, the CS in dark blue, the Ubiquitin hydrolase-like domain (UCH-like) in violet and the RNase in pink. The Pan3straight protomer (Pan3s) is in orange and Pan3bent (Pan3b) in yellow. Of note is the apparent conformational flexibility of the UCH-like–RNase module with respect to the rest of complex (less well resolved details; also compare the 2D class averages of Pan2cat-Pan3Δ[1-162] in A). (C) Details of the Pan2cat-Pan3Δ(1-162) model with corresponding density of the 3D reconstruction. Some areas of the reconstruction show details along the best axis to be expected at the nominal global resolution of 4.5 Å contrasting with the anisotropy of the data and the conformational flexibility of the complex. (D) Superposition of a Pan2-Pan3ΔN composite model (gray, based on superposition of the Pan3 dimer of PDB:4czy [Jonas et al., 2014] and PDB:4xr7 [Schäfer et al., 2014]) and the cryoEM based Pan2cat-Pan3Δ(1-162) model. The WD40 domain has a different orientation with respect to the rest of the complex.

**Figure 3:**
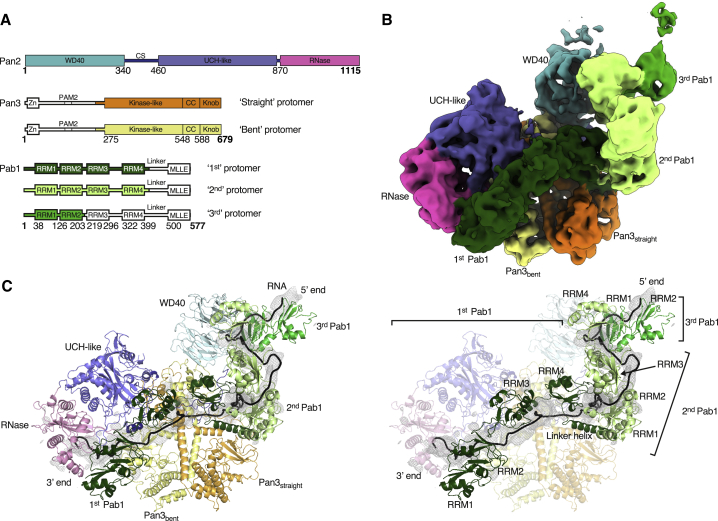
Overall Structure of a 90A RNP-Pan2-Pan3 Complex (A) Schematic representation of the domain organization of Pan2, Pan3, and Pab1. The different protomers and their respective domains visualized in the structure of the 90A RNP-Pan2-Pan3 complex are highlighted in different colors. Absent or untraceable regions are in white. Folded domains are shown as rectangles, and low-complexity sequences (such as the connecting segment CS of Pan2 or the Pab1 linker) are shown as bars. Numbers refer to the domain boundaries. (B) Full cryoEM reconstruction of the Pan2-Pan3-90A RNP complex segmented and colored according to individual protomers or domains (in the case of Pab1 and Pan3 or in the case of Pan2 respectively, colors as in (A), with green showing the poly(A) RNP; see also Figure S2, Figure S3, Figure S4). (C) The corresponding pseudo-atomic model of the Pan2-Pan3-90A RNP complex. The difference density (black mesh) shows the path of the poly(A) RNA. The pseudo-atomic model of the poly(A) RNA defines the overall binding path but cannot be interpreted in detail due to the limited resolution.

**Figure S3:**
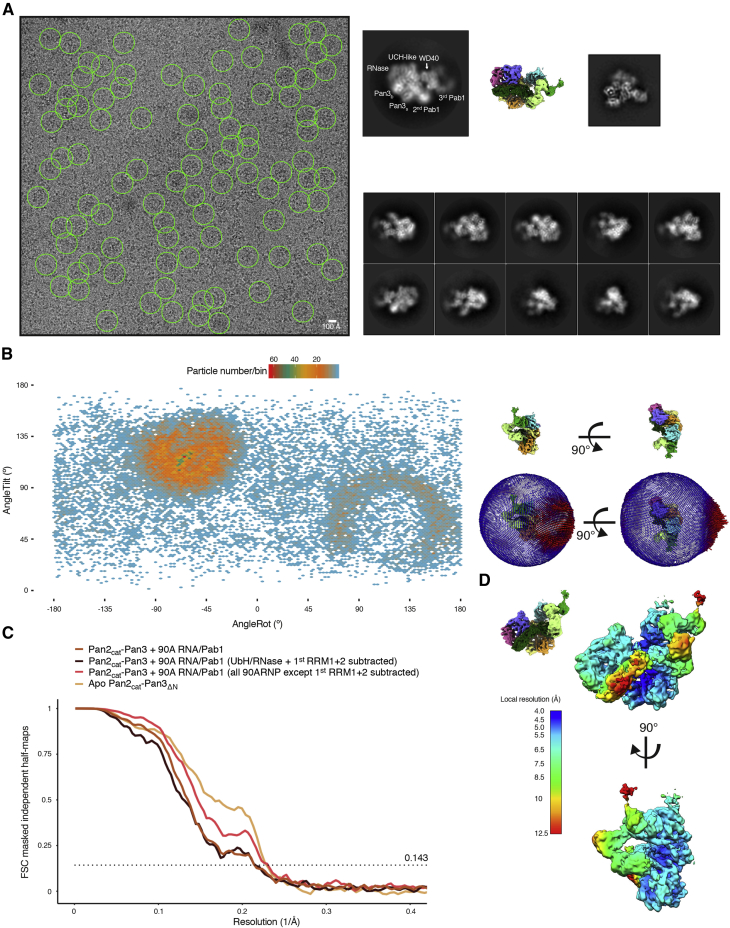
Initial cryo-EM Analysis of the Pan2-Pan3-90A RNP Complex, Related to Figure 3 (A) Representative micrograph (at 0° pre-tilt, on the left-hand side) and reference free 2D class averages (on the right) of the Pan2-Pan3-90A RNP. The scale-bar in the cryo-EM micrograph corresponds to 100 Å and the green circles (260 Å diameter) indicate particles contributing to the initial reconstruction with a nominal global resolution of 7.1 Å (see Figure S4). The 2D class average at the top on the right is contrasted with the 3D reconstruction of the Pan2-Pan3-90A RNP complex and a 2D class average of the Pan2-Pan3 apo data in similar orientations. (B) Angular distribution of particles contributing to the full Pan2-Pan3-90A RNP complex reconstruction. In the panel on the left tilt and rotation angles were plotted against one another for the final 4.8 Å 3D reconstruction (Map 1 in Figure S4). The color of each sampling bin indicates the number of particles in the respective bin. As in the spherical angular distribution representation on the right, bins colored in blue have fewer particles and red ones more (29 165 particles in total). (C) Fourier Shell Correlation (FSC) of masked independent half-maps of the Pan2-Pan3–90A RNP reconstructions used for modeling and structure interpretation (see also Figure S4 for details). According to the gold standard FSC cut off of 0.143 (Rosenthal and Henderson, 2003) the nominal overall resolution of the full Pan2-Pan3 reconstruction is 4.8 Å (Map 1, light brown curve). The reconstruction focused on the RNase/RRM1-RRM2 interaction has a nominal global resolution estimated as 4.5 Å (Map 3, red curve) and that of the reconstruction focusing on the recognition of the Pab1-Pab1 oligomerization interface is 4.7 Å (Map 2, dark brown curve). The FSC of the apo Pan2-Pan3 is also blotted for comparison (sand colored curve). (D) Map of the full Pan2-Pan3–90A RNP colored according to local resolution estimation. The core of Pan2-Pan3 and the Pan2-Pan3–90A/Pab1 interacting regions are resolved at higher resolution whereas parts of the 90A/Pab1 RNP not in contact with the deadenylase complex are less well resolved.

**Figure S4:**
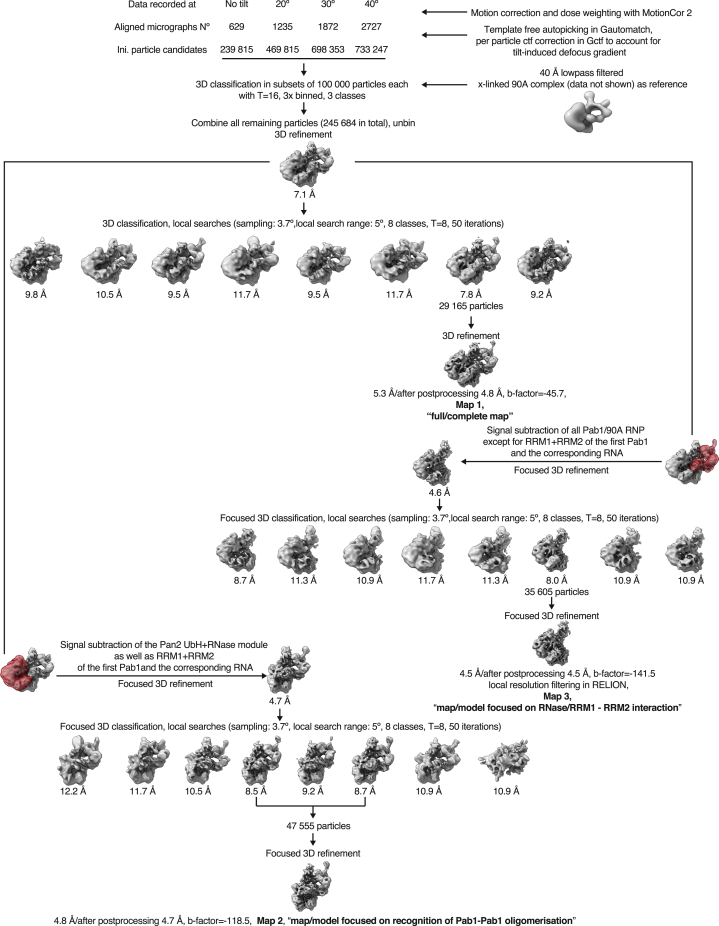
Overview of the cryo-EM Data-Processing Scheme, Related to Figure 3 Particle sorting and classification tree used for 3D reconstruction of the Pan2-Pan3–90A RNP complex. The individual nominal global resolutions are quoted as good proxies for the translational and rotational accuracy of the reconstructions as well as for the level of detail observed in the individual maps. As outlined in the STAR Methods section and mentioned in Figure S3, three individual 3D reconstructions were mainly used for model fitting and structure interpretation. These are the reconstruction of the whole complex at a nominal overall resolution of 4.8 Å (Map 1), the reconstruction after signal subtraction of the Pan2-Pan3/1st Pab1 RRM1 and RRM2 complex at a nominal global resolution of 4.5 Å (Map 3) as well as the reconstruction of the Pan2-Pan3–90A RNP after signal subtraction of the UCH-like–RNase domain and RRM1 and 2 of the 1st Pab1 at a global resolution of 4.7 Å (Map 2). The masks used for signal subtraction are highlighted in red.

We first determined the cryo-EM structure of the full-length Pan2cat-Pan3 deadenylase complex. The reconstruction at a global nominal resolution of ∼4.5 Å showed ordered density for the core of the complex (corresponding to Pan2-Pan3ΔN), whereas the N-terminal region of Pan3 appeared to be disordered (Figure S2). The density allowed us to unambiguously place the known atomic models of the individual domains in Pan2-Pan3 (Jonas et al., 2014, Schäfer et al., 2014) and locally rebuild them where necessary (Figures 3A and S2). We then purified Pan2cat-Pan3 in complex with long poly(A) RNPs for structural analysis. While we purified complexes with either a 70A RNP (containing two Pab1 molecules; Figure 2B, right panel) or a 90A RNP (containing three Pab1 molecules; Figure 2B, left and central panels), the latter yielded better results in the structural analyses. In hindsight, this is consistent with the activity data showing that a longer 90A RNP is a better Pan2-Pan3 substrate than a shorter 70A RNP (Figures 1C and 1D; Table S2). The cryo-EM analyses (Figures S3 and S4) were carried out on a dataset collected with a sample tilting strategy to obtain a more balanced distribution of orientations and to overcome preferred orientation bias (Tan et al., 2017). The resulting 3D reconstruction reached an overall nominal resolution of 4.8 Å (Figure S3C). Features of the deadenylase core appear more rigid and better defined in comparison to the poly(A) RNP, which is more flexible (see also local resolution estimation in Figure S3D; for details on data collection and processing and model building see the STAR Methods section).

Pan2-Pan3 has essentially the same structure in the 90A RNP-bound state compared to the substrate-free state (Figures 3B, 3C, and S2B). Briefly, Pan2 wraps around an intertwined homodimer formed by two Pan3 protomers (Christie et al., 2013, Jonas et al., 2014, Schäfer et al., 2014, Wahle and Stubbs, 2014). The Pan3 homodimer has a pronounced asymmetry stemming from the central coiled coil. The two Pan3 protomers adopt either a straight or a bent conformation, named accordingly as Pan3straight and Pan3bent (Figures 3B, 3C, and S2B). The Pan3 asymmetry is in turn recognized by the Pan2 connecting segment (CS), which positions the N-terminal WD40 domain and the C-terminal ribonuclease module on opposite sides of the Pan3 homodimer. Within the Pan2 ribonuclease module, the ubiquitin C-terminal hydrolase (UCH)-like domain interacts with the knob of Pan3straight and places the RNase adjacent to the pseudokinase of Pan3bent (also labeled as “kinase-like”) (Figures 3B, 3C, and S2B). On the other side, the Pan2 WD40 domain uses a lateral surface of its β-propeller fold to bind the knob of Pan3bent (Figures 3B, 3C, and S2B). Similarly to the substrate-free structure, the 90A RNP-bound reconstruction showed no clearly ordered density for the N-terminal region of Pan3. Although the Pan3 N-terminal region contains two elements that have been implicated in substrate recognition previously (the Zn-finger domain [Wolf et al., 2014] and the PAM2 motif [Siddiqui et al., 2007]), the reconstruction shows that the core complex (i.e., Pan2-Pan3ΔN) directly recognizes the 90A RNP.

### The Periodic Architecture of a 90A RNP when Bound to the Pan2-Pan3 Deadenylase

The 90A RNP forms a long tubular structure that zigzags on the surface of Pan2-Pan3 (Figures 3B, 3C, and 7A). Modeling three RNA-bound Pab1 protomers in this density was aided by specific features of the RNP, including recognizable repeating units and boundaries between the RRM domains of Pab1 (details in the STAR Methods section and quality indicators in Table S1). Structural studies on the human PABPC1 orthologue had shown how RRM1 and RRM2 pack side-by-side to form a bilobal unit that binds RNA in an extended conformation, in an adenosine-specific manner, and with defined polarity (i.e., with the 3′ end of the RNA positioned near the N terminus of RRM1 and the 5′ end near the C terminus of RRM2) (Deo et al., 1999). Using bioinformatic analysis (Kelley et al., 2015), we obtained a similar model of the RRM1-RRM2 module of yeast Pab1, whereas the prediction of RRM3 and RRM4 showed that they are likely to form individual units connected by short helices.

**Figure 4:**
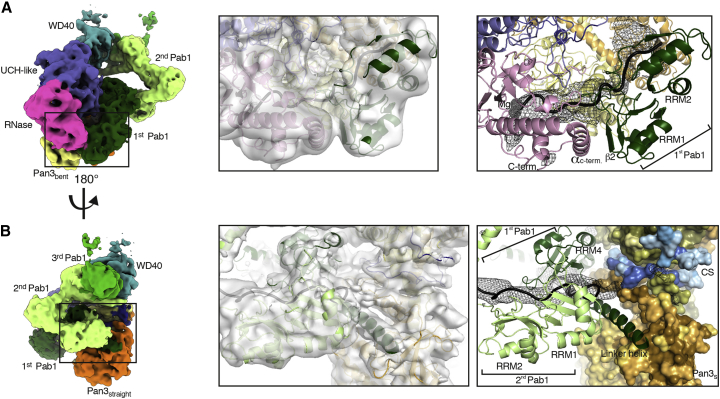
90A RNP Recognition by the Pan2 RNase Domain and the Pan3straight Pseudokinase Domain (A) Views of the interaction between UCH-like-RNase modules of Pan2 (violet and pink, respectively) and the RRM1-RRM2 module of the first Pab1 (dark green). The pseudo-atomic model (right panel) is superposed on the cryo-EM density (central panel). The left panel identifies the overall position of the interface in the context of the reconstruction (shown as segmented density, as in Figure 3B). Difference density for the RNA is shown in mesh representation, in black. The directionality of RNase-Pab1 recognition is fixed by the defined polarity of the poly(A) recognition by RRM1-RRM2 (3′ end at the N terminus, 5′ end at the C terminus) as well as the 3′-to-5′ exonuclease activity of Pan2 (additional details in Figure S5A). (B) Corresponding views of the interaction between the Pan3s pseudokinase domain and the first RNP oligomerization interface (i.e., RRM4-linker helix of the first Pab1 protomer and the RRM1-RRM2 module of the second protomer). Pan3s is shown in a surface representation colored according to evolutionary conservation (dark orange for conserved residues). The RNP contacts the connecting segment (CS) of Pan2, also shown in a surface representation and colored according to conservation (conserved residues in dark blue; see also Figure S5B).

**Figure S5:**
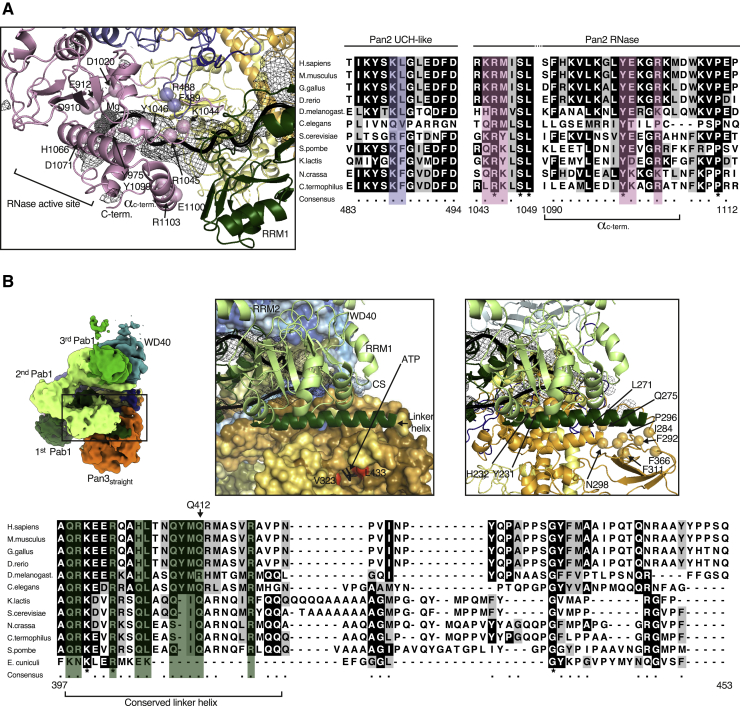
Evolutionarily Conserved Interactions between Pan2-Pan3 and Pab1, Related to Figure 4 (A) Details of the interactions at the 3′ end of the 90A RNP. On the left is a panel corresponding to Figure 4A, with the position of conserved residues from the Pan2 UCH-like domain (violet) and RNase (pink) indicated by spheres. On the right are sequence alignments of the corresponding regions of Pan2 (with conservation shown in blue for the UCH-like domain and pink for the RNase domain). The strongly conserved catalytic residues of the catalytic DEDDh motif around D1020 and D910 are highlighted as is residue Y975 which interacts with the base moiety of AMP in a previously described UCH-like–RNase crystal structure (Schäfer et al., 2014). (B) Details of the interactions at the first Pab1-Pab1 oligomerization interface of the 90A RNP. The panel on the left highlights the overall position of the interface in the context of the reconstruction (corresponding to Figure 4B, after a counterclockwise rotation of 90º). The panel in the middle shows the position of ATP bound to the pseudokinase domain of Pan3straight (from PDB:4bwp, Pan3s shown in surface representation; Christie et al., 2013). In red are evolutionarily conserved ATP-binding residues as reference. The panel on the right lays out the RNP-interacting structural elements of Pan3straight. Spheres indicate the position of conserved residues on Pan3. The lower panel shows the evolutionary conservation of the Pab1 helical segment downstream of RRM4 (the linker helix) and of parts of the (less conserved) rest of the linker.

We started by fitting the RRM1-RRM2 module of the Pab1 protomer located at the 3′ end (i.e., the first protomer to be removed upon deadenylation) into the bilobal density adjacent to the Pan2 RNase domain (Figures 3C and 4A). Here, the conserved N-terminal edge of RRM1 packs against the conserved C-terminal α-helix of Pan2 (Figures 4A and S5A). The position of RRM1-RRM2 orients the 3′ end of the RNA at the entrance of the deadenylase cavity, which is shaped by conserved residues of the RNase and UCH-like domains (Figures 4A and S5A). A difference density calculation indeed shows that the RNA 3′ end extends from RRM1 and enters into the RNase active site (right hand panel of Figure 4A and Figure S5A). C-terminal to RRM2, the density continues in two globular densities where we positioned RRM3 and RRM4 (Figures 3C and 7A). RRM3 appears to be a rather flexible unit, whereas RRM4 engages in extensive interactions. RRM4 is unusual in that it extends into a prominent elongated density that is consistent with the presence of a protruding α-helix (Figure 4B). Bioinformatics predictions (Kelley et al., 2015) also suggest that RRM4 is followed by a long α-helix characterized by conserved positively charged amino acids (residues 398 to 420, hereafter termed “linker helix”) (Figures 4B and S5B). The Pab1 linker helix contacts a conserved surface of the Pan3straight pseudokinase domain, approaching the ATP-binding site (Figures 4B and S5B). On the other side, the linker helix and the RRM4 domain pack against a bilobal density, into which we fitted the RRM1-RRM2 pair of the next, the second Pab1 molecule (Figures 4B and S5B).

**Figure 5:**
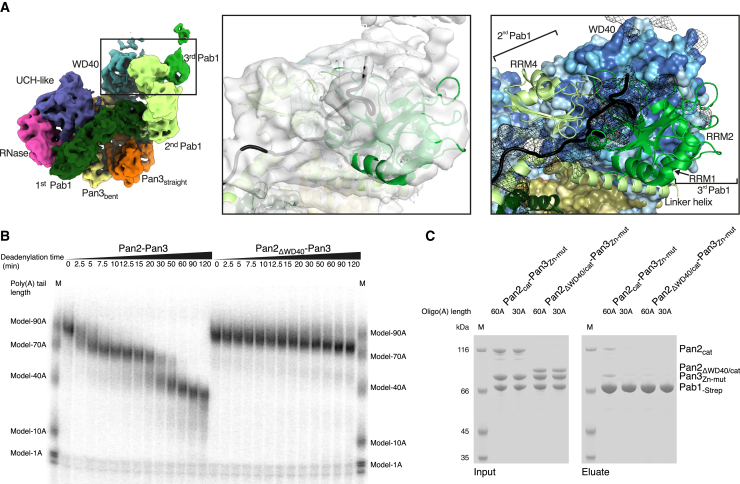
The Pan2 WD40 Domain Senses the Length of the poly(A) RNP (A) Views of the interaction between the Pan2 WD40 domain and the second RNP oligomerization interface (i.e., RRM4-linker helix of the second Pab1 protomer in light green and the RRM1-RRM2 module of the third protomer in green). The Pan2 WD40 domain is shown in a surface representation colored according to evolutionary conservation, where a darker shade of blue represents stronger conservation (Figure S6A for more details). (B) Pan2ΔWD40-Pan3 has strongly reduced deadenylation activity on a yeast 90A RNP. 5′ radioactively labeled model-90A RNA was mixed with Pab1 (in a 1:3 RNA:protein ratio) and incubated with either wild-type Pan2-Pan3 (left) or Pan2ΔWD40-Pan3 (right) over 2 h. At indicated time points, samples were taken and analyzed on a 6% Urea-PAGE followed by phosphorimaging (compare also to Figure S6B). (C) Pan2ΔWD40cat-Pan3Zn-mut (see the STAR Methods section; Figures S6D and S6E) has reduced affinity to a 60A-Pab1 RNP in Strep-Tactin pull-down assays. Both Pan2ΔWD40cat-Pan3Zn-mut and a Pan2cat-Pan3Zn-mut were preys in a Strep-Tactin pull-down with 30A-Pab1-Strep and 60A-Pab1-Strep as bait. The eluate from the Strep-Tactin resin was analyzed on a 4%–12% SDS-PAGE followed by Coomassie staining.

**Figure S6:**
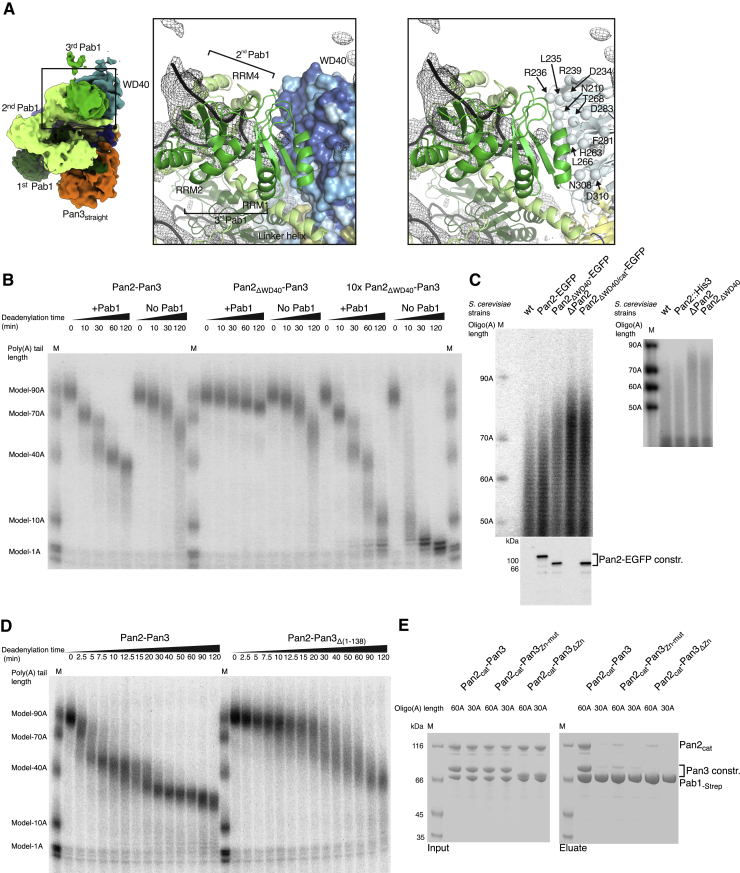
Determinants of Pan2-Pan3 Recruitment to the Poly(A)/RNP, Related to Figure 5 (A) Details of the interactions between the second Pab1-Pab1 oligomerization interface of the 90A RNP and the Pan2 WD40 domain. The panel on the left highlights the overall position of the interface in the context of the reconstruction (shown as segmented density, as in Figure 3B). Conserved surface residues of the WD40 domain in proximity to the Pab1-Pab1 interface are accentuated in a dark blue shade in the surface representation (panel in the middle) and as spheres in the cartoon model on the right. (B) Pan2ΔWD40-Pan3 deadenylation activity is not stimulated by Pab1. 5′ radioactively labeled model-90A RNA was mixed with Pab1 (in a 1:3 RNA:protein ratio,”+Pab1”) and incubated with either wild-type Pan2-Pan3 (left hand side of the gel) or Pan2ΔWD40-Pan3 (right hand side of the gel, equimolar and 10x the amount of wild-type Pan2-Pan3) over a 2 h period. The model-90A RNA in the absence of Pab1 (“no Pab1”) was in parallel also used as substrate in similar deadenylation reactions. At indicated time points samples were taken and analyzed on a 6% Urea-PAGE followed by phosphorimaging. (C) The Pan2 WD40 domain influences poly(A) tail length *in vivo*. The upper panel shows phosphorimages of 8% UREA-PAGE of the pCp labeled, RNase A treated poly(A) isolations from mutant Pan2 yeast strains (Pan2-EGFP tagged strains on the left, S. cerevisiae strains carrying untagged Pan2 variants on the right). In the bottom panel is the respective anti-EGFP western blot. (D) Removal of the Pan3 N terminus has a limited effect on deadenylation activity of a yeast 90A RNP. 5′ radioactively labeled model-90A RNA was mixed with Pab1 (in a 1:3 RNA:protein ratio) and incubated with either wild-type Pan2-Pan3 (left hand side of the gel) or Pan2-Pan3Δ (1–138) (right hand side of the gel) over a 2 h period. At indicated time points samples were taken and analyzed on a 6% Urea-PAGE followed by phosphorimaging. (E) The Pan3 N-terminal Zn-fingers do not contribute to the poly(A) length dependency of the Pan2-Pan3–poly(A)/Pab1 interaction but increase affinity for the poly(A)/Pab1 RNP in co-precipitation experiments. Pan2cat-Pan3, Pan2cat-Pan3Zn-mut (in which the three Cys and one His coordinating the Zn ion have been mutated to Ser or Ala respectively) as well as Pan2cat-Pan3ΔZn (which is Pan2cat-Pan3Δ[1-74]) were preys in a Strep-Tactin pull-down with 30A/Pab1-Strep and 60A/Pab1-Strep as bait. The eluate off the Strep-Tactin resin was analysed on a 4%–12% SDS-PAGE followed by Coomassie staining.

**Figure S7:**
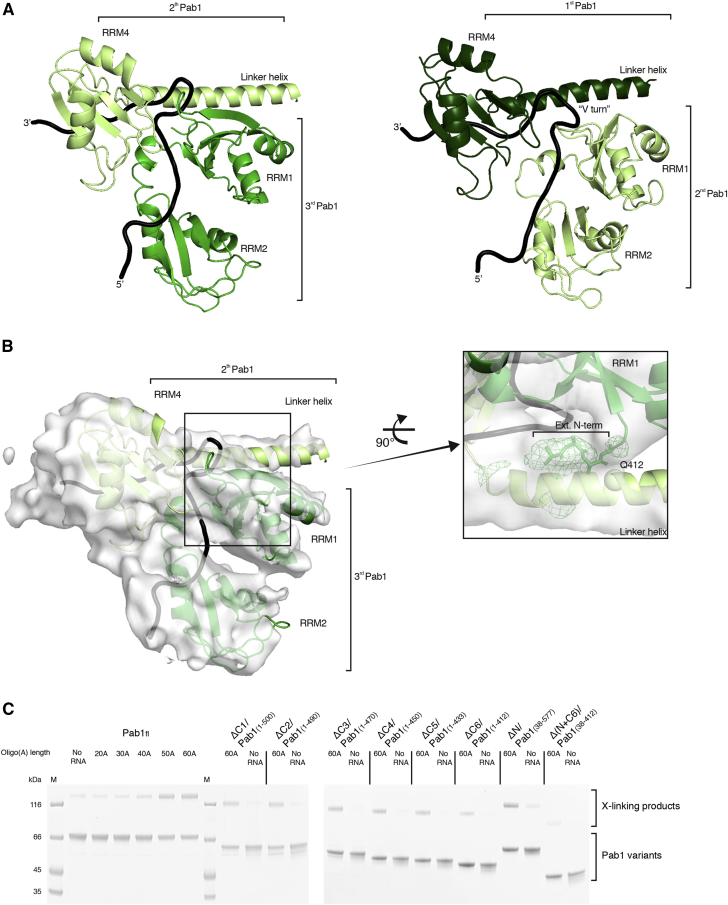
The Pab1-Pab1 Oligomerization Interface, Related to Figure 5 (A) Juxtaposition of the first (right-hand panel) and the second (left-hand panel) Pab1-Pab1 oligomerization interface in cartoon representation and similar orientations. The 1st Pab1 is colored in dark green, the 2nd Pab1 in light green and the 3rd Pab1 in green and the RNA in black. The overall architecture of the two interfaces is very similar. The linker helix of the more 3′ Pab1 interacts in both cases with RRM1 of the more 5′ Pab1 forcing the RNA into a sharp roughly 110° turn (“V turn,” compare Figure 7A). The directionality of Pab1-Pab1 oligomerization is fixed by the defined polarity with which the poly(A) RNA binds the RRM1-RRM2 unit. This interaction provides most the RNA-binding affinity in the context of the full-length protein. (B) Details of the 2nd Pab1-Pab1 interaction interface. The panel on the left shows the model with the corresponding area of density. On the right is a close up of the linker helix-RRM1 interface in density. A sphere indicates the approximate position of Q412. The main chain N-terminal to residue 38 of RRM1 (the leader sequence) is emphasized in licorice representation. Difference density for this N-terminal part is shown in green in a radius of 20 Å around the site. (C) Ruthenium-based photo-crosslinking assay (photo-induced crosslinking of unmodified proteins or PICUP) of Pab1 constructs in the presence of varying length of RNA. On the left is the assay with full-length Pab1 in the presence of increasing length of oligo (A) followed by the experiment with C-terminal and N-terminal truncations of Pab1 in the presence and absence of 60A RNA. Crosslinking is most severely impaired in the construct lacking all residues N-terminal of amino acid 38 and all residues C-terminal to amino acid 412 in the linker helix. The approximate position of Q412 is indicated by a sphere in (B) and highlighted in the multiple sequence alignment in Figure S5B.

At the oligomerization interface between the first and second Pab1, the RNP density adopts a sharp V-shaped turn (Figures 3B, 3C, and 7A). The cryoEM density then continues with another tight turn (∼90º), where two globular features were docked with the RRM3 and RRM4 domains of the second Pab1. Also in this protomer, a long tubular density, consistent with the linker helix, extends from RRM4 and contacts the RRM1-RRM2 pair of the third Pab1 molecule (i.e., the protomer at the 5′ end) (Figures 3C, 5A, S6A, and S7A). At the oligomerization interface between the second and third Pab1, the RNP interacts with the WD40 domain of Pan2 at a conserved surface of the base of the β-propeller (Figures 5A and S6A). The RNP density ends with a partially ordered density that is not in direct contact with the deadenylase core (Figure 3B). Here, the rest of the third Pab1 appears to extend flexibly into solvent. Notably, the portion of Pab1 downstream of the linker helix is disordered in all three Pab1 protomers, suggesting that the MLLE domain can be freely accessible to its binding partners (Xie et al., 2014) in the context of the poly(A) RNP. Also accessible are the other known protein-protein interaction surfaces in the Pan2cat-Pan3-90A RNP assembly, namely the Pan3-binding site for the microRNA regulatory cofactor TNRC6/GW182 (Christie et al., 2013) and the Pab1-binding site for the translation initiation factor eIF4G (Safaee et al., 2012). In general, the zigzagging arrangement of the poly(A) RNP that we observe in the cryo-EM reconstruction is consistent with the RNA-binding footprint of the poly(A)-binding protein (Webster et al., 2018), with the hinges between RRM domains that had been inferred by single-molecule studies (Lee et al., 2014) and with the overall worm-like appearance in negative-stain studies (Sawazaki et al., 2018).

### Molecular Basis and Recognition of Pab-Pab1 Oligomerization Interfaces

The density features corresponding to the two Pab1-Pab1 oligomerization interfaces in the cryo-EM structure are remarkably similar. The linker helix that extends from RRM4 of one Pab1 protomer interacts with RRM1 of the adjacent one (Figure S7A), including a segment of the sequence preceding the RRM1 fold (referred to as leader sequence; Figure S7B). We tested the cryo-EM oligomerization model biochemically by purifying mutants of yeast Pab1 with N-terminal (ΔN) and C-terminal (ΔC) deletions and analyzing them in a ruthenium-based photo-crosslinking assay (photo-induced crosslinking of unmodified proteins, PICUP) (Vollers et al., 2005) (Figure S7C). In the presence of a 60A RNA, we observed a decrease of intermolecular crosslinking between the Pab1 1-433 (ΔC5) and 1-412 (ΔC6) truncation mutants (Figure S7C). The difference between these two constructs is exactly the C-terminal part of the linker helix (residues 397 to 421; see Figure S5B). Further removal of the N-terminal leader sequence effectively impaired Pab1 oligomerization (Pab1 38-412, Δ[N+C6] truncation mutant; Figure S7C), in line with the structural analysis above and consistent with previous data showing the deleterious effects of RRM1 and linker mutants on deadenylation *in vivo* (Simón and Séraphin, 2007, Yao et al., 2007, Zhang et al., 2013). This mechanism rationalizes the conundrum of how the linker can play an important role in mediating Pab1-Pab1 intermolecular interactions while being the least conserved part of the molecule, as the helix downstream of RRM4 is the only evolutionarily conserved part of the linker (see sequence alignment in Figure S5B).

**Figure 6:**
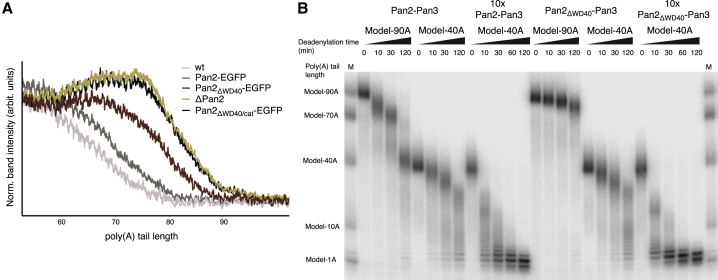
The Pan2 WD40 Domain Distinguishes Long and Short poly(A) RNPs (A) Long poly(A) tails accumulate in S. cerevisiae strains carrying Pan2ΔWD40, ΔPan2, or Pan2ΔWD40/cat. Densitogram of a phosphorimage from an 8% UREA-PAGE separating 3′ labeled poly(A) preparations from haploid yeast strains with indicated variants in the Pan2 genomic ORF. See Figure S6C for the phosphorimage and anti-EGFP western blot. (B) Only 90A RNP, but not 40A RNP, deadenylation is dependent on the WD40 domain of Pan2. 5′ radioactively labeled model-90A and model-40A RNAs were mixed with Pab1 (in a 1:3 and 1:1 RNA:protein ratio, respectively) and incubated with either wild-type Pan2-Pan3 (left) or Pan2ΔWD40-Pan3 (right) at 2.5 or 25 nM (“10×”) final concentration over 2 h. At indicated time points, samples were taken and analyzed on a 6% Urea-PAGE followed by phosphorimaging.

In the structure, the two Pab1 oligomerization interfaces are recognized by Pan2-Pan3 at sites of known functional importance *in vivo*: in proximity to the ATP-binding site of Pan3 (Figures 4B and S5B) (Christie et al., 2013) and the WD40 domain of Pan2 (Figures 5A and S6A) (Jonas et al., 2014). The structural analysis suggests that the Pan2 WD40 domain is crucial for recognizing the second Pab1-Pab1 dimerization interface (between the second and third Pab1 protomers) and thus key to recognizing the length of the poly(A) tail. We tested this mechanism by assessing the properties and effects of a mutant complex lacking the WD40 domain (Pan2ΔWD40-Pan3) both *in vitro* and in cells. As a control, deletion of the Zinc finger domain of Pan3 (which does not contribute to the interaction with poly(A) RNP interaction in our cryo-EM structure) had a comparatively modest effect on overall 90A RNP deadenylation (Figures S6D and S6E). In contrast, a recombinant Pan2ΔWD40-Pan3 mutant was severely impaired in its ability to deadenylate a 90A RNP (Figure 5B) and was, furthermore, not stimulated by the presence of Pab1 (Figure S6B). Also consistent with the structural model, deadenylation of a 40A RNP was not affected by the WD40 deletion. The Pan2ΔWD40-Pan3 mutant showed the same degradation activity on a 40A RNP as the wild-type protein, even at elevated enzyme concentrations (Figure 6B).

To understand the impact of the Pan2 WD40 domain in cells, we used a gene replacement strategy in a BY4743 haploid yeast strain in which the chromosomal copy of PAN2 had been deleted (labeled ΔPan2 in Figures 6A and S6C). We integrated GFP-tagged wild-type PAN2 or pan2-ΔWD40 at the endogenous locus (with or without the D1020A inactivating mutation; labeled Pan2-EGFP, Pan2ΔWD40-EGFP and Pan2ΔWD40cat-EGFP in Figures 6A and S6C; for untagged versions, see right hand panel in Figure S6C). We first confirmed that the mutant proteins were expressed at levels comparable to the wild-type protein, as judged by western blot (bottom panel of Figure S6C). We then isolated and radioactively labeled mRNA poly(A) tails from these strains by 3′ pCp labeling and RNase A digest to assess the effect of the WD40 domain on poly(A) tail length *in vivo*. As controls, strains carrying a deletion of Pan2 or carrying the D1020A active site mutation accumulated longer poly(A) tails than wild-type strains. This is in line with previous reports on a Pan3 deletion (Brown and Sachs, 1998, Mangus et al., 2004) (Figures 6A and S6C). Importantly, the Pan2ΔWD40 strains also led to the accumulation of long poly(A) tails, similar to the ΔPan2 and the Pan2ΔWD40/cat strains (Figures 6A and S6C). This is consistent with the prediction from our structural and *in vitro* results that the WD40 domain of Pan2 is crucial for the preferential recognition of long poly(A) RNPs.

## Discussion

**Figure 7:**
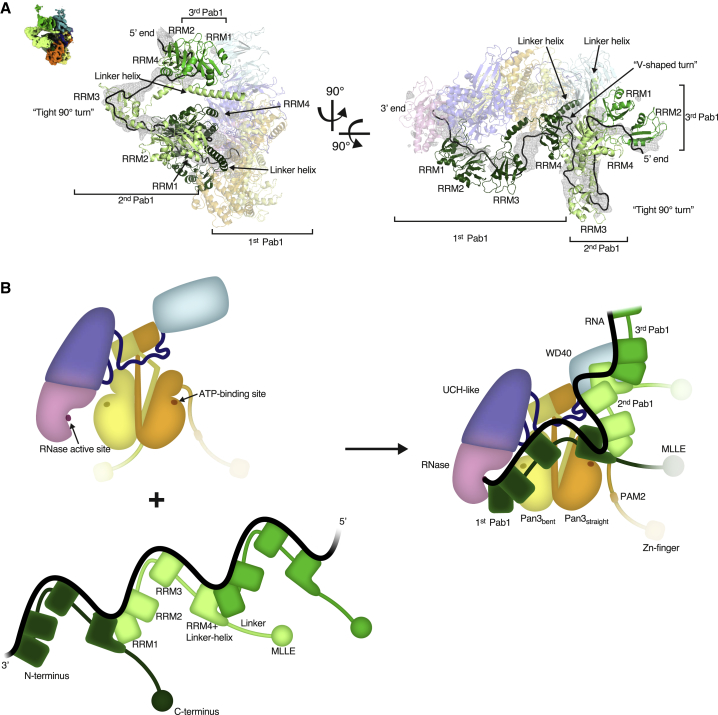
Model of the First mRNA Deadenylation Step (A) Side and top views of the pseudo-atomic model of the Pan2-Pan3-90A RNP complex. The difference density (black mesh) shows the path of the poly(A) RNA (compare also Figure 3C). (B) The schematic depicts how Pan2-Pan3 carries out the first deadenylation step by recognizing a long poly(A) RNP, shortening and eventually releasing it when it contains only a single Pab1 protomer. The drawing recapitulates the features observed in the cryo-EM structure and integrates them with the results from the biochemical characterization.

The cryo-EM reconstruction of the Pan2-Pan3-90A-Pab1 RNP complex we report here provides the first mechanistic insights into the structure of a cytoplasmic poly(A) RNP. The poly(A) RNP can be described as a sequence of arches (Figure 7B), each formed by the four RRMs of a single Pab1 protomer and by the corresponding tract of bound poly(A) tail. The C-terminal MLLE domain of Pab1 appears to be flexibly attached to the arch scaffold via the Pab1 linker. The linker is mostly disordered, with the exception of the long linker helix that extends from the last RRM. With hindsight, this helix would probably be better considered as part of an unusually elongated RRM4 domain. The individual arches of the 90A RNP are connected at the Pab1-Pab1 interfaces, where the elongated RRM4 domain (i.e., RRM4 + linker helix) of a Pab1 protomer interacts with the RRM1-RRM2 module of the adjacent protomer, aligned 3′-5′ along the poly(A) chain. When bound to Pan2-Pan3, the 90A RNP arches differ in size and shape: the first arch at the 3′ end is low and wide, whereas the second one is sharp-pointed and narrow. This conformation appears to be dictated by two properties: the presence of a flexible joint at the RRM3 domain and the relative position of the interacting Pan2-Pan3 surfaces.

The three Pab1 molecules bound on the 90A poly(A) tail interact with Pan2-Pan3 mainly via the two oligomerization interfaces. The most 3′ Pab1-Pab1 oligomerization interface is recognized near one of the ATP-binding sites of Pan3, whereas the more 5′ Pab1-Pab1 oligomerization interface is recognized by the Pan2 WD40 domain. In addition, the RRM1 domain of the most 3′ Pab1 protomer interacts with the RNase domain of Pan2 and threads the RNA into the active site. This intricate substrate recognition mode explains the preference of the Pan2-Pan3 deadenylase for long poly(A) RNPs. Together with our *in vitro* data, these structural observations offer a glimpse into how the substrate-binding affinity of the deadenylase complex progressively decreases as the poly(A) RNP is shortened and Pab1 protomers are progressively removed. We can envision a possible mechanism for how Pan2-Pan3 moves into the poly(A) tail and displaces Pab1 protomers in a stepwise fashion. Starting from the most 3′ Pab1 protomer, once deadenylation prompts RRM1-RRM2 (the high RNA-binding affinity domains) to detach from RNA, the deadenylase swiftly removes RRM3 and RRM4 (the low-affinity RNA-binding domains) and degrades the corresponding portion of RNA. When Pan2-Pan3 encounters the first Pab1-Pab1 oligomerization interface and the RRM1-RRM2 domains of the second Pab1, it temporarily stalls as it slowly peels off the high-RNA-binding-affinity domains of Pab1 from the poly(A) tail. These kinetic blocks correspond to the stepwise accumulation of deadenylated fragments. The Pab1-Pab1 oligomerization interfaces might thus play both a stimulatory role by being the main structural feature recognized by Pan2-Pan3 and simultaneously an inhibitory role functioning as the kinetic obstacles in the reaction. Conversely, the Pan2-Pan3 affinity and deadenylation activity on poly(A) RNPs depends on the number of Pab1-Pab1 oligomerization interfaces present in the poly(A) RNP and, thus, the length of the poly(A) tail. When two Pab1 protomers remain on the shortened poly(A) tail, a single contact point between a Pab1-Pab1 oligomerization interface remains at the Pan3 site, decreasing the ability of the deadenylase to bind the substrate. Once a single Pab1 is left, the poly(A) RNP is inefficiently bound and deadenylated by Pan2-Pan3.

Recent transcriptome-wide studies have shown that the modal length of poly(A) tails in yeast peaks at ∼30 nucleotides (Chang et al., 2014, Subtelny et al., 2014), which is long enough to accommodate one Pab1 molecule. These global measurements thus appear to correlate with a steady-state situation in which the mRNPs have been deadenylated by Pan2-Pan3, are in a translation-competent state, and have not yet been committed to translational silencing and turnover by Ccr4-Not. In human cells, the modal length of poly(A) tails is longer (Eckmann et al., 2011), suggesting that the handover to CCR4-NOT generally occurs when the poly(A) tail has been shortened to contain two PABPC1 protomers. As human PAN2-PAN3, at least *in vitro*, appears to have similar biochemical properties to the yeast orthologue (Figure S1D), the different steady-state lengths of poly(A) tails in human cells may reflect different properties of human CCR4-NOT and/or the presence of longer poly(A) tails on nascent transcripts. More generally, we envision that the modularity and flexibility of the cytoplasmic poly(A) RNP arches could be similarly exploited in the recognition of other decay or translation factors and that additional features, for example, in the 3′ UTR, could influence the structure of poly(A) RNPs of particular transcripts and potentially modulate deadenylation.

## STAR★Methods

### Key Resources Table

**Table:** 

REAGENT or RESOURCE	SOURCE	IDENTIFIER
**Antibodies**		

α-gfp monoclonal antibody	Santa Cruz	sc-9996
goat α-mouse HRP polyclonal antibody	Bio-Rad	172-1011
Bacterial and Virus Strains		
Rosetta (DE3) *E.coli* strain	Merck Millipore	70954
BL21 (DE3) *E.coli* strain	NEB	C2527l

**Chemicals, Peptides, and Recombinant Proteins**		

*S. cerevisiae* Pab1 fl and derivatives	Schäfer et al., 2014; this paper	N/A
*S. cerevisiae* Pan2-Pan3 and derivatives	Schäfer et al., 2014; this paper	N/A
*S. cerevisiae* Ccr4-Caf1-Not1	Basquin et al., 2012	N/A
*H. sapiens* PAN2, PAN3 and PABPC1	This paper	N/A
Phusion High-Fidelity PCR Master Mix	NEB	M0531S
All used restriction enzymes	NEB	N/A
T7-polymerase	Produced in house	N/A
DNaseI (RNase-free)	Thermo Fisher Scientific	AM2222
T4 RNA ligase 1	NEB	M0204S
RNase A (Biochemistry Grade)	Ambion	AM2274
CIP	NEB	M0290S
T4 Polynucleotide Kinase	NEB	M0201S
Proteinase K	NEB	P8107S
ATP [γ-32P]	PerkinElmer	NEG002A100UC
pCp [5’-32P]	PerkinElmer	NEG019A250UC
acid phenol:chloroform	Ambion	AM9720
phenol:chloroform:isoamylalkohol (25:24:1)	Ambion	AM9730
chloroform:isoamylalkohol	SIGMA-ALDRICH	25668
NuPAGE 4-12% Bis-Tris protein gels	Thermo Fisher Scientific	NP0336BOX
8–16% stain-free protein SDS-PAGE gels	Bio-Rad	4568106
Sf-900 II SFM insect cell medium	Thermo Fisher Scientific	10902-104
Amersham ECL prime detection reagent	GE Healthcare	RPN2232

**Deposited Data**		

EMDB	This paper	EMD: 4728
PDB	This paper	PDB: 6R5K

**Experimental Models: Cell Lines**		

*Trichoplusia ni* Hi5 cells	Thermo Fisher Scientific	B85502
*Spodoptera frugiperda* Sf21 cells	Thermo Fisher Scientific	11497013
*S. cerevisiae* BY4743 strain	Euroscarf	http://www.euroscarf.de/index.php?name=News

**Experimental Models: Organisms/Strains**		

isogenic to *S. cerevisiae* BY4741	This paper	WT
*pan2-*EGFP*::kanMX4* in *S. cerevisiae* BY4741 background	This paper	Pan2-EGFP
(Δ1-340*)-pan2-*EGFP*::kanMX4* in *S. cerevisiae* BY4741 background	This paper	Pan2_ΔWD40_-EGFP
(Δ1-340*)-pan2-*D1020A-EGFP*::kanMX4* in *S. cerevisiae* BY4741 background	This paper	Pan2_ΔWD40/cat_-EGFP
*Δpan2*::*klURA3* in *S. cerevisiae* BY4741 background	This paper	ΔPan2
*Pan2::His3MX6* in *S. cerevisiae* BY4741 background	This paper	Pan2::His3
(Δ1-340*)-pan2::His3MX6* in *S. cerevisiae* BY4741 background	This paper	Pan2_ΔWD40_

**Oligonucleotides**		

homo-oligomeric poly(A) RNAs of defined length	ELLA Biotech	N/A

**Recombinant DNA**		

ORFs cloned from genomic DNA of *S.cerevisiae* BY4743 strain and human IMAGE clones	Schäfer et al., 2014; http://www.imageconsortium.org/; this paper	N/A
pcDNA-3.1 (+)	Thermo Fisher Scientific	V79020

**Software and Algorithms**		

MotionCor2	Zheng et al., 2017	https://msg.ucsf.edu/em/software/motioncor2.html
Gctf	Zhang, 2016	https://www.mrc-lmb.cam.ac.uk/kzhang/Gctf/
Gautomatch	N/A	https://www.mrc-lmb.cam.ac.uk/kzhang/Gautomatch/
RELION3.0	Zivanov et al., 2018	https://github.com/3dem/relion
sxviper	Penczek et al., 1994	http://sphire.mpg.de/
phenix.real_space_refine	Afonine et al., 2018b	https://www.phenix-online.org/
phenix.mtriage	Afonine et al., 2018a	https://www.phenix-online.org/
Coot	Emsley et al., 2010	https://www2.mrc-lmb.cam.ac.uk/Personal/pemsley/coot/
PyMOL2	N/A	https://pymol.org/2/
ChimeraX	Goddard et al., 2018	https://www.cgl.ucsf.edu/chimerax/
ConSurf 2016	Ashkenazy et al., 2016	http://consurf.tau.ac.il/2016/
ImageJ	N/A	https://fiji.sc/#
Tidyverse	N/A	https://www.tidyverse.org/

**Other**		

Quantifoil holey carbon grids	Quantifoil	N/A
Superdex200 16/60 column	GE Healthcare	28-9893-35
Superose 6 3.2/300 column	GE Healthcare	29-0915-98
Superdex75 16/60 column	GE Healthcare	17-1068-01
Mono Q 5/50 GL column	GE Healthcare	17-5166-01
Strep-Tactin Sepharose	IBA lifescience	2-1201-002
Glutathion Sepharose 4 Fast Flow	GE Healthcare	17-5132-01
HIS-Select HF Nickel Affinity Gel	Sigma-Aldrich	H0537-25ML
Heparin Sepharose 6 Fast Flow	GE Healthcare	17-0998-01
GFPtrap agarose beads	Chromotek	Gta-20
Sepharose 4b	GE Healthcare	17-0120-01

### Contact for Reagent and Resource Sharing

Further information and requests for resources and reagents should be directed to and will be fulfilled by the Lead Contact, Elena Conti (conti@biochem.mpg.de).

### Experimental Model and Subject Details

#### Origins of Recombinant ORFs

All S. cerevisiae ORFs of the proteins investigated here were initially cloned from genomic DNA preparations of the S. cerevisiae BY4743 strain grown at 30°C in standard YPD. Human PAN2, PAN3 and PABPC1 were cloned from the respective H. sapiens IMAGE clones (http://www.imageconsortium.org/).

#### Heterologous Protein Expression Systems

Pab1 and PABPC1 full-length and truncation constructs were heterologously expressed in the E. coli expression strain Rosetta DE3. Not1(754-1000) was heterologously expressed in the E. coli expression strain BL21 DE3. Cells were grown to an OD600nm of 1.0 at 37°C in standard TB medium, the temperature reduced to 18°C and expression induced by the addition of IPTG at a final concentration of 250 μM. Cells were harvested 16 h post induction. Both yeast and human recombinant Pan2-Pan3 complexes as well as the core yeast Ccr4-Caf1 nucleases were expressed in the Trichoplusia ni Hi5 cell line. All Pan2-Pan3 complexes were subcloned in the pFL expression vector and co-expressed in insect cells using the Multibac method (Fitzgerald et al., 2006). The Ccr4(110-837)-Caf1(146-433) complex was expressed from the pFastBac DUAL vector (Invitrogen). Cells were transfected with recombinant Baculovirus variants carrying Pan2-Pan3 or Ccr4(110-837)-Caf1(146-433) coding sequences and grown for 48 to 60 h in Sf-900 II SFM Medium (Thermo Fisher Scientific) at 27°C. Recombinant Baculovirus generations were amplified in the Spodoptera frugiperda Sf21 cell line grown in Sf-900 II SFM Medium at 27°C.

#### S. Cerevisiae Strains for RNA Isolation

All S. cerevisiae strains carrying PAN2 variants were constructed by gene replacement in the S. cerevisiae BY4743 strain. For RNA isolations cells were grown in YPD to an OD600nm of 1.0 at 30°C and harvested.

### Method Details

#### Recombinant Protein Expression and RNA Transcription

Recombinant yeast and human Pan2-Pan3 proteins and mutants were expressed in insect cells. All Pan2ΔWD40 variants lack residues 1-339 of Pan2 and in all Pan3Zn-mut constructs Cys 14, Cys 24, and Cys 30 have been mutated to Ser and His 34 to Ala. All other deletion constructs (e.g. Pan3Δ[1–162]) lack residues as indicated. Both yeast and human complexes were purified as described (Schäfer et al., 2014) previously. In brief, after cell lysis by sonication, Pan2-Pan3 complexes were purified via a C-terminal deca-histidine tag on Pan3 using Nickel-based affinity chromatography (IMAC, HIS-Select resin (Sigma-Aldrich) was used for all IMAC steps described in this study), followed by ion exchange (Mono Q 5/50 GL) and gel filtration chromatography (Superdex200 16/60, equilibrated in 20 mM HEPES pH 7.5, 200 mM NaCl, 100 μM MgCl2 and 4 mM DTT). S. cerevisiae Pab1 full-length (fl) protein, its truncated forms as well as H. sapiens PABPC1 were subcloned into in-house expression vectors (pEC-A-3C-GST) and were expressed in Rosetta E. coli strains (Merck Millipore). Pab1 and its variants as well as PABPC1 were purified via a N-terminal GST-tag using GSH affinity chromatography. After removal of the GST-tag via 3C protease on-column cleavage, the proteins were further purified via a Heparin column and polished on a gel filtration chromatography column (Superdex200 16/60, equilibrated in 20 mM HEPES/NaOH pH7.5, 200 mM NaCl, 100 μM MgCl2 and 4 mM DTT). The S. cerevisiae Caf1-Ccr4-Not1 core complex (in the text abbreviated to Caf1-Ccr4) was expressed and purified as described (Basquin et al., 2012). Not1(754-1000) was expressed as a fusion construct with a cleavable SUMO-His tag at the N terminus. After IMAC, tag cleavage and a Heparin chromatography step, the protein was purified over a size exclusion column (Superdex75 16/60, equilibrated in 20 mM Tris pH 7.5, 250 mM NaCl, 1 mM DTT). The His-tagged Ccr4(110-837)-Caf1(146-433) complex was purified via consecutive IMAC, Heparin and size exclusion chromatography (Superdex200 16/60, equilibrated in 20 mM Tris pH 7.5, 250 mM NaCl, 1 mM DTT) steps. The S. cerevisiae Caf1-Ccr4-Not1 core complex (in the text abbreviated to Caf1-Ccr4) was reconstituted by mixing purified Not1(754-1000) and Ccr4(110-837)-Caf1(146-433) in equimolar amounts and removing subunit excess via a size exclusion chromatography column (Superdex200 16/60, equilibrated in 20 mM Tris pH 7.5, 250 mM NaCl, 1 mM DTT). Model RNA substrate with poly(A) tails of different lengths were constructed by cloning oligo(A) DNA pieces of defined length into the ApaI site of the pcDNA-3.1 plasmid (Thermo Scientific, Invitrogen). These oligo(A) DNA pieces contain a NsiI at the 3′ end of the sense strand. NsiI digestion leaves a single Thymidine at the 5′ end of the antisense strand ensuring homooligomeric poly(A) tails at the 3′ end of the model substrates. For T7-polymerase-based *in vitro* run off transcriptions the vectors were linearized with NsiI (NEB) and the fragment containing the MCS and the poly(A) tail were isolated using TBE-agarose gel electrophoresis. The T7-polymerase reaction was carried out for 4h at 37°C (40 mM Tris/HCl pH 8.0, 28 mM MgCl2, 0.01% (v/v) Triton X-100, 5 mM DTT, 1 mM Spermidine). The DNA template was removed from the reaction by DNaseI digest and the resulting product (123 bp+poly(A) tail of variable length) purified using phenol/chloroform extraction followed by ethanol precipitation. For the deadenylation reactions, the *in vitro* transcribed RNAs were CIP treated and 32P -5′-labeled via phosphorylation by T4-Polynucleotide Kinase and ATP [γ-32P] as phosphate donor according to manufacturers’ instructions.

#### Size-Exclusion Chromatography Assays

For the size-exclusion chromatography (SEC) assays in Figure 2B, purified Pab1 (36 μM final concentration in the case of 90A, 24 μM in the case of 70A and either 36 μM (“3x 30A/Pab1”), 24 μM (“2x 30A/Pab1”) or 12 μM (“Pab1” and “Pab1/30A”) final concentration in the case of 30A was mixed with the respective RNA (90A oligo RNA at 12 μM final concentration, 70A oligo RNA at 12 μM, 30A oligo RNA at either 36 μM [“3x 30A/Pab1”], 24 μM [“2x 30A/Pab1”] or 12 μM [“Pab1/30A”]) final concentration and incubated at 4°C for 30 min (all oligo(A) RNAs were procured from Ella Biotech). Pan2cat-Pan3 (12 μM final concentration) was added to these reactions and again incubated for 15 min at 4°C in a total injection volume of 30 μl in SEC buffer (20 mM HEPES/NaOH pH 7.5, 50 mM NaCl, 100 mM potassium acetate, 5 mM magnesium acetate, 2 mM DTT). Complex formation was assayed by comparing the retention volumes in SEC on a Superose 6 3.2/300 column (GE Healthcare). Composition of the SEC peak fractions were analyzed by SDS-PAGE on 4%–12% NuPAGE gradient gels (Thermo Fisher Scientific) and visualized by Coomassie staining. For better comparison, each individual chromatogram was normalized by its maximal absorption value and plotted in R using the tidyverse collection of R packages.

#### Pull-Down Assays

For the pull-down assay described in Figure 2C, Pab1 at a final concentration of 24 μM was mixed with the respective oligo(A) RNAs (20A, 40A, 60A) at a final concentration of 12 μM in pull-down buffer 1 (20 mM HEPES/NaOH pH 7.5, 50 mM NaCl, 100 mM potassium acetate, 5 mM magnesium acetate, 4 mM DTT, cOmplete Protease inhibitor (Roche), 0.01% (w/v) NP40) and incubated for 30 min on ice. His-tagged Pan2cat-Pan3 was added to a final concentration of 12 μM and the reaction again incubated for 30 min on ice. The sample were then incubated with IMAC resin (HIS-Select resin, Sigma-Aldrich) which interacts with the 10×His-tag on the C terminus of Pan3 for 1 h at 4°C under agitation. Free proteins were washed off the resin by four successive wash steps using 1ml of pull-down buffer 2 at each step (20 mM HEPES/NaOH pH 7.5, 50 mM NaCl, 100 mM potassium acetate, 5 mM magnesium acetate, 4 mM DTT, cOmplete Protease inhibitor (Roche), 0.01% (w/v) NP40, 30 mM Imidazole pH 7.5). Samples were eluted in elution buffer (identical to pull-down buffer 2, with 500 mM Imidazole instead of 30 mM). The sample were mixed with SDS-loading loading buffer analyzed by SDS-PAGE on 4%–12% NuPAGE gradient gels (Thermo Fisher Scientific) and visualized by Coomassie staining.

For the pull-down assay described in Figure 2D, Pab1-eCFP-StrepII at a final concentration of 12 μM was mixed with the respective oligo(A) RNAs (20A, 30A, 40A, 50A, 60A, 70A) at a final concentration of 12 μM and untagged Pab1 at a final concentration of 12 μM in pull-down buffer 1 (20 mM HEPES/NaOH pH 7.5, 50 mM NaCl, 100 mM potassium acetate, 5 mM magnesium acetate, 4 mM DTT, Complete Protease inhibitor (Roche), 0.01% (w/v) NP40) and incubated for 30 min on ice. The samples were then incubated with Strep-Tactin resin (IBA lifesciences) for 1 h at 4°C under agitation. Free proteins were cleared off the resin by four consecutive wash steps using 1 mL of pull-down buffer 1 for each wash step. Samples were eluted in elution buffer (pull-down buffer 1 supplemented with 10 mM buffered Desthiobiotin). The sample were mixed with SDS-loading buffer analyzed by SDS-PAGE on 4%–12% NuPAGE gradient gels (Thermo Fisher Scientific) and visualized by Coomassie staining. The protocol for the pull-down described in Figure S1C is very similar to what has been described above. In contrast, however, an N-terminal GST fusion of Pab1 was used as bait in this case, GSH affinity resin (GSH Sepharose 4 fast flow, GE Healthcare) to pull-down proteins and pull-down buffer 1 supplemented with 30 mM buffered, reduced GSH to elute proteins off the resin.

For the pull-down assay described in Figures 5C and S6D, Pab1-StrepII at a final concentration of 24 μM was mixed with the respective oligo(A) RNAs (30A or 60A) at a final concentration of 12 μM in pull-down buffer 3 (20 mM HEPES/NaOH pH 7.5, 100 mM NaCl, 2 mM MgCl2, 4 mM DTT, cOmplete Protease inhibitor (Roche), 0.01% (w/v) NP40) and incubated for 30 min on ice. The respective Pan2cat-Pan3 constructs were added to a final concentration of 12 μM and the reactions again incubated for 30 min on ice. The samples were then incubated with Strep-Tactin resin (IBA lifesciences) for 1 h at 4°C under agitation. Free proteins were washed off the resin by two sequential wash steps using 500 μl of pull-down buffer 3 each time. Samples were eluted in elution buffer (pull-down buffer 3 supplemented with 10 mM buffered Desthiobiotin). The samples were mixed with SDS-loading loading buffer analyzed by SDS-PAGE on 4%–12% NuPAGE gradient gels (Thermo Fisher Scientific) and visualized by Coomassie staining.

#### Deadenylation Assays

Deadenylation reactions were carried out at 30°C for 10 min in a buffer containing 50 mM HEPES/NaOH, pH 7.5, 50 mM potassium acetate, 1 mM magnesium diacetate, 0.1 mg/ml bovine serum albumin and 1 mM DTT. The poly(A) RNP was reconstituted by mixing the respective, 5′ radioactively labeled RNA at a final concentration of 50 nM with Pab1 at a final concentration of 150 nM in case of the reactions with the 90A-model substrate, 100 nM in case of the reactions with the 70A-model substrate and 50nM in the case of the reactions with a 40A-model substrate. This mixture was incubated for 30 min at 4°C and the reaction started by the addition of the respective Pan2-Pan3 complex at a final concentration of 2.5 nM if not indicated otherwise and/or Caf1-Ccr4-Not1 at a final concentration of 250 nM if applicable. At indicated time points, 5 μl of each sample was removed and the deadenylation reaction was immediately stopped by the addition of 5 μl stop buffer composed of 50 mM EDTA and 0.1% (w/v) SDS. The proteins were removed by Proteinase K (NEB) digest and diluted with 30 μl loading dye containing 10 mM EDTA, 0.1% (w/v) bromophenol blue and 0.1% (w/v) xylene cyanol FF in formamide and boiled at 95°C for 4 min. The products were resolved on a 6% polyacrylamide gel containing 7 M Urea. As molecular weight markers we used individual or mixed 5′-labeled model RNAs with poly(A) tails of indicated length. Products were visualized by phosphorimaging after overnight exposure at –80°C. The densitometry in Figure 1D was performed in R using the tidyverse collection of R packages. To determine the half-lives of the intermediates of the reaction (Table S2) decay of each product was quantified via densitometry in ImageJ. A total of three deadenylation time courses were densitometrically quantified (including Figure 1C). The raw data for each time point were normalized by the maximal signal and the mean normalized signal for each time-point calculated. An exponential function of the form $f\left(t\right)=a*e^{-St}$ where a is the signal at time point 0, S is the decay rate and t is the time in seconds was subsequently fitted to the data. In the case of the intermediates, Model-70A and Model-40A, time point 0 was chosen as the time-point where most of the specific product had accumulated (i.e., where the densitometric signal was the highest). The resulting model allowed the determination of the half-lives and their mean standard deviation intervals for the Model-90A substrates and its two main intermediates, the Model-70A and the Model-40A substrate.

#### Crosslinking Assays

For the zero length cross-linking assay (PICUP; Vollers et al., 2005) described in Figure S7C, Pab1 variants alone or as Pab1/oligo(A) RNPs (with 20A, 30A, 40A, 50A, or 60A oligo RNAs, reconstituted by incubation for 30 min on ice, final concentration of Pab1 in all samples 1 μM) were mixed with Tris(2,2-bipyridyl) dichlororuthenium(II)hexahydrate and APS at final concentrations of 0.5 mM and 2 mM respectively in cross-linking buffer (20 mM HEPES/NaOH pH 7.5, 250 mM NaCl, 100 mM K(CH3COO), 5 mM Mg(CH3COO)2). The reaction was induced by a 1 sec flash of a 452 nm led light and cross-linking was quenched by the addition of SDS-loading buffer. Samples were subsequently analyzed by SDS-PAGE on 4%–12% NuPAGE gradient gels (Thermo Fisher Scientific) and visualized via Coomassie staining.

#### Saccharomyces Cerevisiae Strain Construction

The generated strains are based on the S. cerevisiae BY4743 strain (Euroscarf, #Y20000). Pan2 knockout strains were created according to standard protocols in yeast genetics described for example in (Janke et al., 2004). Briefly, a linear dsDNA fragment encoding the klURA3 ORF with flaking sequences homologues to the pan2 genomic ORF was transformed into the diploid BY4743 strain and selected for autotrophy accordingly. Sporulation was induced in a strain which carried the selection marker and also tested positively in a diagnostic PCR for the disruption of the pan2 ORF. Tetrads of this strain were analyzed and the haploid pan2 knockout strain was used for subsequent strain construction.

Linear DNA fragments harboring the desired pan2 variants were generated by Gibson assembly (NEBS2611S; see Gibson et al., 2009). For this, pan2 constructs were amplified from previously generated expression plasmids (Schäfer et al., 2014 and this study) and joined with a selection marker (HIS3MX6) amplified from a well-established plasmid toolkit for yeast genetics (Janke et al., 2004). The resulting linear DNA fragments were transformed into the haploid pan2 deletion strain and the deletion cassette replaced by the desired construct/mutant of pan2. The transformations of the strains followed a method described in (Gietz and Schiestl, 2007). Briefly, we harvested cells from 50 mL YPD culture grown to a density of OD600nm ∼ 1 AU. The cells were washed three times and resuspended in H2O to a final volume of approximately 1 mL 100 μl of cells were used per transformation. Each aliquot of cells was mixed with the respective transformation mix each consisting of 240 μl PEG3350 (50% w/v), 36 μl 1 M lithium acetate, 10 μl of single stranded herring sperm DNA (10mg/ml), 64 μl of H2O and 10 μl of the individual double stranded DNA product generated by Gibson assembly. The transformation reactions were incubated at 28°C overnight without agitation before platting on agar plates. This was necessary since the pan2 knockout strain does not responded well to elevated temperatures as required in the 42°C heat shock treatment. G418R, FOAR and HIS- strains were selected according to the auxotrophy/resistance they confer and the mutations of pan2 were subsequently confirmed by sequencing. Tagged versions of the pan2 variants with EGFP::kanMX4 were generated by replacing the HIS3MX6 cassette to facilitate detection of the protein products by immunoblotting (see below).

#### Immunodetection of pan2-eGFP

An overnight culture was diluted to OD600nm = 0.2 AU and grown in YPAD at 28°C. Cells corresponding to 15 OD600nm were harvested, washed once in ddH2O and resuspended in 1.5 mL lysis buffer (20mM Tris-HCl pH 7.5, 150 mM NaCl, 0.5 mM EDTA, 0.1% NP-40, 1 tablet Roche cOmplete protease inhibitor per 50 mL buffer, benzonase 95 U/ml final activity). The cell suspension was lysed by the glass bead method using a Precellys (Bertin) homogenizer fitted with a Cryolys (Bertin) to maintain a temperature of 2–4°C. Cells were lysed by eight consecutive cell rupture cycles à 40 s at 9500 rpm with 45 s long interspersed breaks. The suspension was subsequently centrifuged/cleared in a microcentrifuge for 10 min at 16,000 × g and 4°C. The resulting lysates were preincubated for 45 min with 25 μl Sepharose 4b beads (GE Lifesciences) at 4°C. The beads were spun down and the supernatant was incubated for 2.5 h with 25 μl GFPtrap agarose beads (Chromotek, gta-20) at 4°C under agitation. The beads were washed twice in lysis buffer and finally resuspended in 50 μl 2× SDS buffer. The samples were boiled for 5 min at 96°C and spun down for 5 min/16 000 g. 10 μl of these samples were separated on 8%–16% stain-free protein SDS-PAGE gels (Bio-Rad, #4568106) and subsequently blotted onto a nitrocellulose membrane with 400mA for 1.5h at 4°C. The western blot was developed on an iBind device (Life Technologies). As first antibody we used a 1:80 dilution of the α-gfp monoclonal antibody (Santa Cruz sc-9996) in 2 mL iBind solution and as second antibody a 1:4000 dilution of the goat α-mouse HRP polyclonal antibody (Bio-Rad 172-1011) in 2 mL iBind solution. The blot was developed using Amersham ECL prime detection reagent (GE Lifesciences RPN2232) and subsequently imaged on a GE Lifesciences LAS4000.

#### Poly(A) Isolation from *S. cerevisiae* Strains

Bulk poly(A) isolations and 3′-radioactive labeling via pCp were performed similar to what has been described before (Brown et al., 1996). Yeast cultures were inoculated from fresh plates (not older than 3 days) and grown to an OD600nm of ∼1.20 mL of these cultures were harvested by centrifugation at 3600 × g for 2 min at 4°C, washed once in ice cold, RNase-free water, pelleted again as above, frozen in liquid nitrogen and stored at −80°C.

For isolation of total RNA, cells were thawed in 700 μl extraction buffer (20 mM Tris-HCl pH 8.0, 10 mM EDTA pH 8.0, 300 mM NaCl) supplemented with 80 μl 10% SDS and mixed with 600 μl acid phenol:chloroform preheated to 65°C. The samples were vortexed for 30 s, incubated for 2 min at 65°C. The aqueous phase was removed, and the hot phenol extraction was repeated once as described above. The aqueous phase was removed and extracted either twice with phenol:chloroform:isoamylalcohol (25:24:1; in the case of the EGFP tagged strains) or once with phenol:chloroform:isoamylalcohol and once with chloroform:isoamylalcohol. Total RNA was precipitated by addition of 80 μl 3M potassium acetate pH 5.3 followed by ethanol precipitation. Pellets were resuspended in 40 μl RNase free water and 800 μg total RNA from each strain were 3′-radioactive labeling by 5′-32P pCp using T4 RNA ligase 1 (NEB) for 1 h at 37°C. The labeled samples were digested with RNase A (Ambion) for 1 h at 37°C. The radioactively labeled poly(A) tails were extracted with phenol:chloroform:isoamylalcohol and ethanol precipitated for 1 h at −20°C. The dried RNA pellets were resuspended in 40 μl loading dye containing 10 mM EDTA, 0.1% (w/v) bromophenol blue and 0.1% (w/v) xylene cyanol FF in formamide and boiled at 95°C for 4 min. The products were resolved on an 8% polyacrylamide gel containing 7 M Urea. In addition to the RNA extracts from the yeast strains carrying Pan2 variants, a mix of 90A, 70A, 60A, and 50A oligo(A) RNA was processed in an identical way as positive control and used as marker on the UREA-PAGE. The densitometry in Figure 6A was performed in R using the tidyverse collection of R packages. In brief the raw intensity reads from the lane-by-lane densitometry were calibrated with the marker and normalized by the maximum intensity in each sample. Two individual lanes of each sample were averaged and the background signal subtracted.

#### cryo-EM Grid Preparation and Imaging

The Pan2-Pan3–90A/Pab1 RNP was reconstituted as described for the size exclusion assays. The sample was purified on a Superose 6 3.2/300 (GE Healthcare) and peak fractions were adjusted to an OD280nm of 1.1 (typical OD260nm/OD280nm of ∼1.7 were indicative of RNA presence), n-octyl-β-D-glucoside was added to a final concentration of 0.04% (v/v) and 4 μl of this sample was applied to glow discharged (2.2×10−1 mbar for 2x20sec) Quantifoil holey carbon grids (R2/1,200 mesh, Quantifoil). The grids were plunge vitrified into a liquid ethane/propane mix using a Vitrobot Mark IV at 4°C and 95% humidity. Cryo-EM data were collected on a FEI Titan Krios microscope operated at 300 kV, equipped with a post-column GIF and a K2 Summit direct detector operating in counting mode. A total of 6,463 movies were recorded at a nominal magnification of 130,000x that corresponds to 1.06 Å/pixel at the specimen level. Initial screening data indicated severe preferred orientation with only one dominant view apparent in 2D and 3D processing trials (data not shown here). Since no other approach tested overcame this problem we collected the data in 4 groups: with 0 degrees (629 micrographs), with 20 degrees (1235 micrographs), with 30 degrees (1872 micrographs) and with 40 degrees (2727 micrographs) stage pretilt (Tan et al., 2017). For the 0, 20, and 30 degrees pretilt data collection we imaged with a total exposure of 51 e-/Å2 at the specimen level evenly distributed over 40 frames during 8 s. The movies of the 40 degrees group were collected with a total exposure of 52 e-/Å2 evenly distributed over 80 frames again during 8 s. As a preset target global defocus range we used 0.5 to 3.5 μm. The sample preparation and data collection strategies for the apo Pan2-Pan3 samples were very similar except no n-octyl-β-D-glucoside was used for grid preparation. These data were collected with a Volta phase plate and a total exposure of 30 e-/Å2 spread over 30 frames and 6 sec. No stage pretilt was employed. The target defocus ranged between 0.25 and 0.75 μm.

#### cryo-EM Data Processing

MotionCor2 was used to correct for beam-induced sample motions and radiation damage taking into account the pretilt of the stage (Zheng et al., 2017). The summed and dose-weighted micrographs were used for further processing. Particles were selected using Gautomatch (https://www.mrc-lmb.cam.ac.uk/kzhang/Gautomatch/). CTF parameters were determined and per-particle refinement using Gctf was performed to account for the defocus gradient across micrographs in the pretilted data (Zhang, 2016). If not stated otherwise all further processing was carried out in RELION-2.1 or RELION-3.0 (Zivanov et al., 2018). After particle extraction, the per-micrograph CTF information in the particle .star file was replaced by the per-particle CTF information using an R script. A total of 239 815 particle candidates at 0 degrees, 469 815 particle candidates at 20 degrees, 698 353 particle candidates at 30 degrees and 733 247 particle candidates at 40 degrees pretilt were initially selected and cleaned using 3D classification. Initial optimization of classification efficiency resulted in best sorting being obtained with a Bayesian fudge factor (T value) of 16. The starting model for the initial round of 3D classification was a 40 Å low-pass filtered model of a cross-linked Pan2-Pan3–Pab1/90A RNP particle for which the initial starting model had been generated with sxviper (Penczek et al., 1994) (not discussed here). The resulting cleaned dataset of 245 684 particles reached a nominal global resolution of 7.1 Å in 3D refinement. We quote the global resolution estimates of all obtained reconstructions good proxies for the overall relative quality of the individual reconstructions, fully acknowledging the differences in local resolution estimates from higher in the Pan2-Pan3 core to lower in the 90A/Pab1 RNP as well as the anisotropy of the data (compare Figures S3D and S4). Since many details of Pan2-Pan3, the 90A/Pab1 RNP and the interaction between both remain elusive in the before mentioned cryoEM map we followed a three-pronged approach to further elucidate structural details of the complex. We classified the data in 8 classes with finer sampling and a T value of 8. The class revealing the finest details, having the highest nominal overall resolution and the most precise rotational and translational accuracy reconstructed to a nominal global resolution of 4.8 Å after 3D refinement and automatic negative B-factor weighting as well as high resolution noise substitution in the postprocessing routine of RELION (Map 1 in Figure S4 and Table S1, 29 165 particles). The resulting map is depicted in Figures 3B and 3C (as map versus model difference density), in all other overview images as segmented density and in Figure 7A (as difference density). The angular distribution of particles contributing to this map is shown in Figure S3B and the FSC curve of the masked independent half-maps in Figure S3C. The rotation versus tilt angle plot in Figure S3B was created by binning the angular assignments of all particles contributing to this reconstruction in 3° by 1.5° bins respectively followed by plotting the resulting distribution using the tidyverse collection of R packages. The local resolution estimate in Figure S3D was calculated with the local resolution routine implemented in RELION-3.0 (Zivanov et al., 2018). Since the density for RRM1 and RRM2 of the 1st Pab1 is less well defined compared to the rest of the poly(A) RNP and the UCH-like–RNase module of Pan2-Pan3 appears flexible compared to the rest of the complex in the substrate free reconstruction we subtracted the signal for those parts from the particles contributing to the initial 7.1 Å 3D refinement. After focused classification the two best classes were combined, 3D refined and postprocessed and resulted in a reconstruction with a nominal global resolution of 4.7 Å (Map 2 in Figure S4 and Table S1). The corresponding map and model are depicted in Figure 4B, Figure 5A, Figures S5B and S6A (in the case of Figures S5B and S6A only as difference density) and Figure S7. To elucidate the interaction of the Pan2 RNase with RRM1 and RRM2 of the 1st Pab1, we subtracted the rest of the poly(A)/Pab1 RNP from the particles in the initial 7.1 Å 3D refinement. Further processing was carried out as described above and in Figure S4 (Map 3 in Figure S4 and Table S1). The resulting map and model are the basis for Figure 4A and Figure S5A (as difference density).

The data of the substrate free Pan2cat-Pan3Δ(1-162) sample were processed using a similar approach as outlined above with the exception that we first filtered the micrographs by discarding those with a maximal resolution estimate in the CTF estimation of worse than 4.5 Å. The extracted particles were cleaned by 2D classification (see Figure S2) and only then further processed in 3D classification and 3D refinement.

#### Modeling and Density Fitting

In a first step we constructed a model for the substrate free reconstruction of Pan2-Pan3. This served as the basis for the interpretation of the three 3D reconstructions of the Pan2-Pan3–90A-Pab1 RNP. After positioning the Pan2-Pan3 model into the densities, individual parts of the Pab1-RNA model were constructed for the two cryoEM densities where we had subtracted parts of the RNP. Finally, the two partial models were combined and the resulting consensus model fitted to the 3D reconstruction of the full complex. We identified 10 small globular domains as RRMs in the density of the poly(A) RNP during this process. We used a priori knowledge of how RRMs, in particular the Pab1 RRMs, interact with RNA to guide the modeling of the Pab1-RNA RNP (Deo et al., 1999, Maris et al., 2005).

In detail, for the substrate free Pan2-Pan3 structure we rigid body fitted the crystal structure of Pan2 UCH-like–RNase module (PDB: 4q8H) and chain B and C (Pan3 dimer) as well as the CS region of chain A (Pan2) of the crystal structure of the core S. cerevisiae Pan2-Pan3 (PDB: 4xr7; Schäfer et al., 2014). A homology model of the WD40 built by PHYRE2 (Kelley et al., 2015) served as starting model for this part of the complex. Comparing the resulting fit to the available crystal structures of the Pan2 WD40 domain from other species (Jonas et al., 2014) validated the positioning of the model for this domain. We locally rebuilt specific parts of the complex such as the connection of the Pan2 CS to the WD40 domain.

In the case of the Pan2-Pan3–poly(A)RNP we first fitted the reconstruction focused on the RNase/Pab1 recognition. After placing the apo Pan2-Pan3 structure, we fitted a homology model based on the crystal structure of the human RRM1 and RRM2 in complex with RNA to the corresponding area of density (PDB: 1cvj; Deo et al., 1999). Subsequently we improved the model in this area by positioning individual homology models of RRM1 and 2 into the respective densities. Since calculation of a map versus model difference density clearly indicated the extension of the RNA into the active site of Pan2 we elongate said molecule along that path.

For the reconstruction where we had subtracted the UCH-like–RNase module and RRM1 and RRM2 of the first Pab1 we again placed the model of Pan2-Pan3 first. We identified 7 (of the 10 total) globular domains as well as 2 long helices in the 90A/RNP density. 4 of the 7 globular domains form into two compact modules we identified as RRM1 and RRM2 of the 2nd and 3rd Pab1. Globular densities that protruded into the above-mentioned 2 long helices precede both. Additionally, since both of these RRM-helix assemblies were the 4th globular feature in a repeating array of domains we identified them as RRM4 and the linker helix of the 1st and the 2nd Pab1 respectively. The remaining globular density at the pronounced kink (“tight 90° turn” in Figure 7A) of the poly(A)-Pab1 RNP between the kinase-like domain of Pan3straight and the Pan2 WD40 domain was identified as RRM3 of the 2nd Pab1. We rigid body fitted RRM homology models as well as predicted helices into each of these assigned features. The linker regions between the individual RRMs are, as has been experimentally confirmed for the RRM1-RRM2 case, predicted to have a propensity to form α-helices. Difference density calculation between the resulting model and the map revealed both the path of the RNA (depicted for example in Figure 3C) as well as those linker segments connecting the RRMs. In areas with ambiguity concerning RNA and RRM linker density we identified the denser feature as RNA (due to the stronger signal of the Phosphate backbone) and/or used geometric constrains (length of linker region, orientation of RNA binding residues on RRMs etc.) for guidance.

The model of the full complex was subsequently constructed by combining Pan2-Pan3 and the individual parts of the 90A-Pab1 RNP. The least well resolved area of the full reconstruction and most ambiguous part of the model is the globular density of RRM3 of the 1st Pab1. Here we connected RRM1 and RRM2 of the 1st Pab1 from the RNase/RRM1-RRM2 interaction model with RRM4 of the 2nd Pab1 of the model focused on recognition of Pab1-Pab1 oligomerization. This resulted in the overall model of the Pan2-Pan3–Pab1/90A RNP. The resulting final model and the cryoEM map of the full Pan2-Pan3–RNP are in good agreement as determined by the correlation coefficients (CC) of as well as the FSC between both (see Table S1).

For all reconstructions/models, rigid body fits were performed in USCF Chimera (Pettersen et al., 2004). The models were manually adjusted in Coot (Emsley et al., 2010) and real space refined in phenix.refine (Afonine et al., 2018b). Progress in modeling was monitored via the map-to-model correlation coefficients and map versus model FSCs (see Table S1). Structure figures were created in PyMOL2 and ChimeraX (Goddard et al., 2018) and the conservation of Pan2-Pan3 was blotted on the model using the ConSurf 2016 server (Ashkenazy et al., 2016).

### Quantification and Statistical Analysis

#### Quantitation of *In Vitro* Deadenylation Assays

The quantitation shown in Figure 1D was performed in R using the tidyverse collection of R packages. The phosphorimage of the deadenylation assay shown in Figure 1C was converted to a data matrix where each entry corresponds to the intensity value of one pixel of the phosphorimage. This matrix was split vertically according to the individual lanes of the gel. For each resulting lane matrix, the average of each row was calculated. This resulted in one vector per lane containing the average values of intensity along the migration direction of the gel of the particular lane (see Figure 1D panel on the left). The peaks of these average intensity distributions for each individual size marker lane were identified. These peak values were used to calibrate migration behavior in the gel with poly(A) tail lengths of the model substrate. Subsequently the maximum of the intensity distributions for each deadenylation time-point was identified. The respective poly(A) tail length was calculated using the calibration curve. The resulting plot is shown in Figure 1D panel on the right.

To determine the half-lives of the intermediates of the deadenylation reaction (Figure S1A and Table S2) decay of each intermediate was quantified in ImageJ. A total of three phosphorimages of deadenylation time courses were quantified (including Figure 1C). The gels were horizontally quantified with the gel quantification tool of ImageJ to follow the decay of the clearly identifiable Model-90A, Model-70A and Model-40A intermediates. This quantification resulted in average intensity distributions for each deadenylation intermediate on each phosphorimage along the time axis of the experiments. The peaks in these intensity distributions correspond to the signals for the respective intermediate at specific time points in the deadenylation reaction. The areas of these peaks for each intermediate and time point were calculated in ImageJ and used for further analysis. These raw data were normalized by the maximal signal in the respective average intensity distribution (to compensate for differences in total signal, exposure etc. between phosphorimages). The mean normalized signal for each time-point and deadenylation intermediate was calculated. An exponential function of the form $f\left(t\right)=a*e^{-St}$ where a is the signal at time point 0, S is the decay rate and t is the time in seconds was subsequently fitted to the data. In the case of the two intermediates the Model-70A and the Model-40A the time point 0 was chosen as the time-point where most of the specific product had accumulated (i.e. where the densitometric signal was the highest). The resulting model allowed the determination of the half-lives and their mean standard deviation intervals for each intermediate (in Figure S1A vertical lines represent the standard deviation interval; the functions fitted to determine the half-lives of the respective model poly(A) RNPs (see Table S2) are represented as lines).

#### Quantitation of Poly(A) Isolations from S. Cerevisiae Strains

The quantification in Figure 6A was performed in R using the tidyverse collection of R packages. The raw phosphorimage shown in Figure S6C was quantified as described for the quantitation of the *in vitro* deadenylation assays. To correct for differences in signal strength between individual poly(A) isolations we normalized each individual intensity distributions by the maximum intensity in the respective sample. Two individual lanes of each strain were averaged and the average background signal subtracted. The resulting poly(A) tail distributions was plotted and is shown in Figure 6A.

### Data and Software Availability

The accession number for the cryo-EM density maps is Electron Microscopy Data Bank: EMD: 4728. The accession number for the atomic model is Protein Data Bank (PDB): 6R5K.
