# Supplementary material for: Molecular Basis for poly(A) RNP Architecture and Recognition by the Pan2-Pan3 Deadenylase
Source: Cell. 2019 May 30;177(6):1619–1631.e21. doi: 10.1016/j.cell.2019.04.013 (PMC6547884; doi:10.1016/j.cell.2019.04.013)
Supplement: Document S1. Tables S1 and S2 [file mmc1.docx]

**Supplemental item titles and legends**

**Table S1. Related to Figure 3**

**Cryo electron microscopy data collection summary, processing statistics and model quality indicators**

| **Cryo electron microscopy data collection** |  | | |
| --- | --- | --- | --- |
| Microscope | FEI Titan Krios GII | | |
| Voltage (kV) | 300 | | |
| Camera | Gatan K2-Summit | | |
| Energy Filter | Gatan Quantum-LS (GIF) | | |
| Pixel size (Å/pix) | 1.059 | | |
| Preset target global defocus range (μm) | 0.5 - 3.5 | | |
|  | | | |
| **3D reconstruction** | **Map 1,**  **full Pan2-Pan3–Pab1/90A RNP** | **Map 2,**  **focus on Pab1-Pab1 oligomerisation recognition** | **Map 3,**  **focus on RNase/**  **RRM1-RRM2** |
| Number of movies | 6463 | | |
| Per stage pretilt angle | 629 (0**°**), 1235 (20**°**), 1872 (30**°**), 2727 (40**°)** | | |
| Initially selected  particle candidates | 239 815 (0**°**), 469 815 (20**°**), 698 353 (30**°**), 733247 (40**°)** | | |
| Final number of particles | 29 165 | 47 555 | 35 605 |
| Resolution _FSC independent halfmaps_ (Å)**^a^** | 4.8 | 4.7 | 4.5 |
| Local resolution range (Å) | 4.0-13 | not determined | not determined |
| Sharpening B-factor (Å^2^) | -45.7 | -118.5 | -141.5 |
|  | | | |
| **Refinement** |  | | |
| No atoms | 23 521 | 17 034 | 16 323 |
| Residues (protein+RNA) | 2932 | 2119 | 2095 |
| Ligands | 2 (Mg) | / | 2 (Mg) |
| CC_box_, CC_mask_, CC_volume_**^b^** | 0.88, 0.72, 0.73 | 0.88, 0.74, 0.73 | 0.82, 0.66, 0.68 |
| CCs of individual chains**^b^** |  | | |
| Pan2, Pan3_s_, Pan3_b_ | 0.75, 0.81, 0.82 |  | |
| 1^st^ Pab1, 2^nd^ Pab1, 3^rd^ Pab1 | 0.79, 0.87, 0.84 |  |  |
| RNA | 0.79 |  |  |
| Resolution _FSC map vs. model@0.143_ (Å)**^b^** | 4.8 | 4.6 | 4.5 |
| r.m.s. deviations |  | | |
| Bond lengths (Å) | 0.005 | 0.007 | 0.008 |
| Bond angles (°) | 1.173 | 1.29 | 1.43 |
| Ramachandran favored (%) | 85.1 | 83.2 | 84.3 |
| Ramachandran gen. allowed (%) | 14.9 | 16.7 | 15.6 |
| Ramachandran disallowed (%) | 0 | 0.1 | 0.15 |
| MolProbity score | 2.3 | 2.4 | 2.3 |
| Clash score | 12.0 | 15.4 | 13.2 |

**^a^**according to the Fourier Shell Correlation (FSC) cut-off criterion of 0.143 defined in (Rosenthal and Henderson, 2003)

**^b^**according to the map-vs.-model Correlation Coefficient definitions in (Afonine et al., 2018a)

**Table S2. Related to Figure 1**

**Half-lives of the 90A model substrate and its deadenylation intermediates in *in vitro* Pan2-Pan3 mediated deadenylation reactions.**

Three phosphorimages of UREA-PAGEs of Pan2-Pan3 mediated deadenylation time courses were densitometrically quantified (similar and including figure 1C; see Figure S1A for the quantitation). The raw data for each time point were normalized by the maximal signal and the mean normalized signal for each time-point calculated. An exponential function of the form $f\left( t \right)={a*e}^{-St}$ where *a* is the signal at time point 0, *S* is the decay constant and *t* is the time in seconds was subsequently fitted to the data. The resulting models allowed the determination of the half-lives and the approximation of mean standard deviation intervals for the Model-90A substrates and its two main intermediates, the Model-70A and the Model-40A substrate.

| **Model substrate in Pan2-Pan3 deadenylation reaction** | half-life [s] (+/- propagated mean standard deviation) |
| --- | --- |
| Model-90A | 135 (+/- 5) |
| Model-70A, intermediate product of Model-90A deadenylation | 384 (+/- 78) |
| Model-40A, intermediate product of Model-90A deadenylation | 990 (+/- 264) |
